# The effects of positive psychology theory in the rehabilitation nursing of Chinese patients with schizophrenia: a systematic review and meta-analysis of randomized controlled trials

**DOI:** 10.3389/fpsyt.2025.1515028

**Published:** 2025-02-19

**Authors:** Yu Hong, Yanjun Huang, Junhong Jiang, Qiuhua Liu, Jing Hu, Wenfei Tan, Jinying Deng, Xintian Wang

**Affiliations:** ^1^ School of Health and Nursing, Guangzhou Huali College, Guangzhou, China; ^2^ Department of Nursing, Guangzhou Zengcheng Health Vocational School, Guangzhou, China

**Keywords:** schizophrenia, positive psychology, treatment as usual (TAU), meta-analysis, randomized controlled trial (RCT)

## Abstract

**Background:**

Schizophrenia is a complex and severe chronic mental disorder characterized by persistent cognitive dysfunction. Particularly in Chinese families, the disability of patients with schizophrenia and the burden on their caregivers are especially heavy, reflecting the profound impact of the disease on both the patients and their families. Positive psychology is a science that focuses on human happiness, strengths, and potential. It originated at the end of the 20th century, advocated by psychologists such as Martin Seligman, aiming to go beyond the traditional psychology’s focus on diseases and deficiencies and instead explore how to help people achieve the best mental state and quality of life. This study aims to conduct a meta-analysis to evaluate the impact of positive psychology interventions on Chinese patients with schizophrenia.

**Objectives:**

This study aims to explore the impact of positive psychology on the wellbeing, positive symptoms, negative symptoms, depressive symptoms, social functioning, social adaptability, and cognitive functions of patients with schizophrenia.

**Methods:**

Literature was retrieved from 11 databases (CNKI, Wan fang Database, VIP Database, CBM Database, PubMed, EMBASE, Cochrane Library, Web of Science, APA PsycINFO, CINAHL, and MEDLINE), with the search period ranging from the inception date to 1 August 2024. Two researchers independently conducted literature reviews, data extraction, and bias risk assessments. The quality of the included studies was assessed using the Cochrane Risk of Bias tool, and meta-analyses were conducted using RevMan 5.3 and Stata 14.0. The continuous outcomes were analyzed by calculating the mean difference (MD) or standardized mean difference (SMD) with 95% confidence intervals (CI) according to whether combining outcomes measured on different scales or not, depending on whether the results measured by different scales were combined.

**Results:**

A total of 54 randomized controlled trials met the criteria for this study. The results showed that positive psychology can significantly improve the wellbeing of Chinese patients with schizophrenia (MD = 0.61, 95% CI = 0.56 to 0.66, *p* < 0.001, *I* ^2^ = 0%), and long-term and mid-term treatments were significantly better than short-term treatments (*p* < 0.001). Positive psychology can significantly improve the psychological health status of Chinese patients with schizophrenia (MD = 43.50, 95% CI = 40.11 to 46.89, *p* < 0.001, *I* ^2^ = 0%), and long-term and mid-term treatments were significantly better than short-term treatments (*p* = 0.004). Positive psychology can significantly improve the positive symptoms of Chinese patients with schizophrenia (SMD = −2.68, 95% CI = −3.53 to −1.84, *p* <0.001, *I* ^2^ = 95%), and long-term and mid-term treatments were significantly better than short-term treatments (*p* < 0.001). Positive psychology can significantly improve the negative symptoms of Chinese patients with schizophrenia (SMD = −2.63, 95% CI = −3.40 to −1.87, *p* < 0.001, *I* ^2^ = 94%), and long-term and mid-term treatments were significantly better than short-term treatments (*p* < 0.001). Positive psychology can significantly improve the social functioning of Chinese patients with schizophrenia (MD = −2.68, 95% CI = −3.26 to −2.10, *p* < 0.001, *I* ^2^ = 94%), and long-term and mid-term treatments were significantly better than short-term treatments (*p* < 0.001). Positive psychology can significantly improve the self-esteem of Chinese patients with schizophrenia (MD = 7.98, 95% CI = −7.53 to 8.42, *p* < 0.001, *I*² = 0%). Positive psychology can significantly improve the social adaptability of Chinese patients with schizophrenia (MD = −8.72, 95% CI = −9.16 to −8.27, *p* < 0.001, *I*² = 0%). Positive psychology can significantly improve the cognitive function of Chinese patients with schizophrenia (MD = 2.38, 95% CI = 1.97 to 2.78, *p* < 0.001, *I* ^2^ = 38%).

**Conclusion:**

Positive psychology has significant effects on enhancing the wellbeing of Chinese patients with schizophrenia. It not only improves the positive and negative symptoms of the disorder but also strengthens social adaptability and cognitive functions. Moreover, positive psychology provides clear benefits in alleviating depressive symptoms among individuals with schizophrenia. Notably, the long-term adherence to positive psychological interventions yields much better treatment outcomes than short-term interventions. Therefore, we recommend the widespread application of positive psychology in clinical treatment.

**Systematic review registration:**

https://www.crd.york.ac.uk/prospero/, identifier CRD42024585178.

## Introduction

Schizophrenia is a complex and severe chronic mental disorder characterized by persistent cognitive dysfunction (1, 2). This disease is marked by high recurrence rates (3), high disability rates (4), and poor prognosis (5), with a condition that often fluctuates and lingers, causing significant suffering to patients both physically and mentally, and can lead to varying degrees of mental disability (6). Schizophrenia typically manifests in young and middle adulthood, with the peak age of onset around 25 years old (7). Approximately 29 million adults worldwide are affected by schizophrenia (8), imposing a heavy burden on public health systems and adversely affecting the quality of life for those affected over the long term (9, 10). In China, the lifetime prevalence of schizophrenia and other mental disorders is as high as 0.75%, with an estimated 10.5 million patients (11).

Data from the CBD (Global Burden of Disease) study shows that from 1990 to 2019, the prevalence of schizophrenia worldwide increased by approximately 66.2% (12). The symptoms of schizophrenia are complex and diverse, mainly including positive symptoms and negative symptoms (13). Positive symptoms encompass hallucinations, delusions, disorganized thinking, abnormal behavior, and disorganized speech, while negative symptoms include emotional blunting, avolition, social withdrawal, poverty of speech, and anhedonia, which severely affect patients’ treatment compliance (14) and social functioning (15). Although antipsychotic medications are currently the mainstream method for treating schizophrenia, the potential side effects they may bring cannot be ignored (16, 17). At the same time, the options for non-pharmacological treatment of schizophrenia are relatively limited, which poses challenges for treatment plans and patient care (18, 19). To improve this situation, clinical treatment is gradually exploring ways to reduce dependence on traditional antipsychotic medications, focusing more on non-pharmacological treatment methods, with the expectation of improving patients’ therapeutic outcomes and quality of life (18).

Positive psychology, proposed by American psychologists Martin Seligman (20), is a science focused on the positive qualities and strengths of human beings. It encourages individuals to discover and cultivate their own advantages and virtues in order to enhance mental health and wellbeing. In the treatment of schizophrenia, the application of positive psychology is gaining attention; it reinforces the positive psychological and social factors of patients, which not only helps alleviate psychotic symptoms but also enhances their sense of happiness and treatment compliance (21). However, despite the potential shown by positive psychology in the treatment of schizophrenia, there is currently a relative lack of systematic reviews and meta-analyses in this field, limiting its application in clinical practice.

The disability of patients with schizophrenia and the burden on their caregivers are particularly significant in Chinese families (10), reflecting the profound impact of the disease on patients and their families. The chronic and unpredictable nature of schizophrenia, along with its associated social dysfunction, brings immense psychological and economic pressure to patients and their families (22). In the field of research on positive psychology interventions for schizophrenia, inconsistent findings have emerged. Some studies have demonstrated that positive psychology significantly enhances patients’ wellbeing and improves depressive symptoms (23, 24). However, other literature indicates that positive psychology does not significantly improve patients’ wellbeing, positive symptoms, negative symptoms, and quality of life (25–27). Thus, there is still a need for comprehensive research to determine its efficacy.

Therefore, this review and meta-analysis aim to systematically evaluate the effectiveness of positive psychology compared to treatment as usual (TAU) on various outcomes such as wellbeing, mental health, positive symptoms, negative symptoms, and social functioning in individuals with schizophrenia across different treatment durations.

## Methods

The original review protocol has been registered and published on PROSPERO (CRD42024585178). To establish the evidence base, this meta-analysis adheres to the Preferred Reporting Items for Systematic Reviews and Meta-analyses (PRISMA) statement (28). Given that all data used in this study are from data sources, ethical approval and informed consent are deemed unnecessary.

### Search strategy

Literature was retrieved from 11 databases (CNKI, Wan fang Database, VIP Database, CBM Database, PubMed, EMBASE, Cochrane Library, Web of Science, APA PsycINFO, CINAHL, and MEDLINE), with the search period ranging from the inception date to 1 August 2024. In addition, we utilized internet search functions, such as www.google.com. We employed the following MeSH terms and keywords: (schizophrenia or dementia praecox or schizophrenic disorder) and (positive psychology or psychology or positive or positive psychology theory) and (randomized controlled trials) and (China or Chinese or Mainland China). Furthermore, we manually checked the reference lists and related commentaries to identify and retrieve other relevant studies. The search strategies for each database are detailed in **Supplementary Appendix S1**.

### Inclusion and exclusion criteria

We followed the PICOS criteria outlined in the “Cochrane Handbook for Systematic Reviews of Interventions” to determine the scope of studies to be included (29). (1) Population: Including Chinese patients aged 18 years and above, diagnosed with schizophrenia, with no gender restrictions. Patients with a history of alcohol or drug dependence or intellectual disabilities are excluded. All participants are from Mainland China, while Hong Kong, Taiwan, and Macau are not included in the scope of this study due to their long-term influence from foreign cultures. (2) Intervention: The experimental group receives treatment based on positive psychology theory, focusing on establishing a positive mindset and cultivating positive psychological qualities in patients. The intervention style can be implemented through face-to-face interviews, relevant lectures, or other means without considering the frequency and duration of the intervention. (3) Comparison: The control group receives no treatment, minimal intervention (such as education, pamphlets), and routine care, without involving positive psychological interventions. (4) Outcome: Wellbeing, mental health, positive symptoms of schizophrenia, negative symptoms of schizophrenia, social function, self-esteem, social adaptability, and cognitive functions. (5) Study design: Inclusion criteria: Only randomized controlled trials (RCTs) were included; patients diagnosed with schizophrenia. Exclusion criteria: Non-RCT research; incorrect population, irrelevant to the results, and failure to report data used for analysis.

### Data extractions

In accordance with the research requirements, we have established a standardized data extraction scheme. Two reviewers (YH and YJH) independently extracted and entered various data in a double-blind manner, and a third reviewer (JHJ) conducted cross-checking. Any discrepancies encountered during this step were resolved through discussion with another reviewer (QHL) to make the final decision. The extracted data include the following: the first author of the literature, publication year, sample size of the experimental and control groups, participants’ age, content of the intervention, intervention plan (duration, frequency, and cycle), outcome indicators, and other relevant data. We determine the effect size by assessing the differences in means between two independent samples. In cases where the standard deviation (SD) is not provided, we estimate it using the standard error (SE), confidence interval (CI), *t*-value, or *p*-value. We make efforts to procure missing data by reaching out to the authors via email (30). Should the authors fail to include the data in the study but offer charts that contain the relevant information, we utilize GetData Digitizer version 2.20 software to extract the required data from these charts. When both unadjusted and adjusted data are presented in a paper, we prioritize the use of adjusted data for our research analysis.

### Quality assessment

According to the guidelines of evidence-based medicine, the risk of bias assessment tool from the Cochrane Systematic Reviews is used (31), where two reviewers (YH and YJH) evaluate the quality of included studies based on seven criteria: random sequence generation, allocation concealment, blinding of participants and personnel, blinding of outcome assessment, incomplete outcome data, selective reporting, and other bias. Any discrepancies are resolved through discussion with a third reviewer (JHJ). In the statistical process, the quality assessment is categorized as follows: studies with six or more criteria are considered to have a low risk of bias; those with three to four criteria have a moderate risk of bias; and those with fewer than three criteria have a high risk of bias.

### Quality of evidence

The assessment of the certainty of evidence is conducted by two reviewers (YH and YJH) based on the Grading of Recommendations Assessment, Development, and Evaluation (GRADE) approach, which evaluates the risk of bias, consistency, indirectness, imprecision, and publication bias. The certainty of the evidence is categorized into four levels (high, moderate, low, and very low) (32, 33).

### Statistical analyses

All analyses were conducted using Review Manager (version 5.3.5; The Cochrane Collaboration, Copenhagen, Denmark) and Stata software (version 14; Stata Corp, Texas, USA). Researchers combined data by summing the mean values and calculating the standard deviations to derive the post-intervention outcomes. For continuous data, we calculated the mean difference (MD) and standardized mean difference (SMD) along with their corresponding 95% CIs, depending on whether the outcomes were measured by the same tool. Researchers will perform statistical tests for heterogeneity (Cochran’s *Q* and *I* ^2^), where the *I* ^2^ statistic is used to assess the degree of heterogeneity. If *I*² ≤ 50% and *P* > 0.1, a fixed-effects model will be used to pool the data; conversely, if *I*² > 50% and *p* < 0.1, a random-effects model will be used (34). Sensitivity analysis by excluding trials one by one and subgroup analysis will be conducted to explore the sources of heterogeneity. We have defined short-term treatment (2–4 weeks), medium-term treatment (6–8 weeks), and long-term treatment (10–12 weeks) groups to explore the effect of intervention duration on outcomes. Finally, we will use the Egger test to assess publication bias and draw a funnel plot using Stata 14.0 software (35).

## Results

### Description of included studies

**Figure 1:**
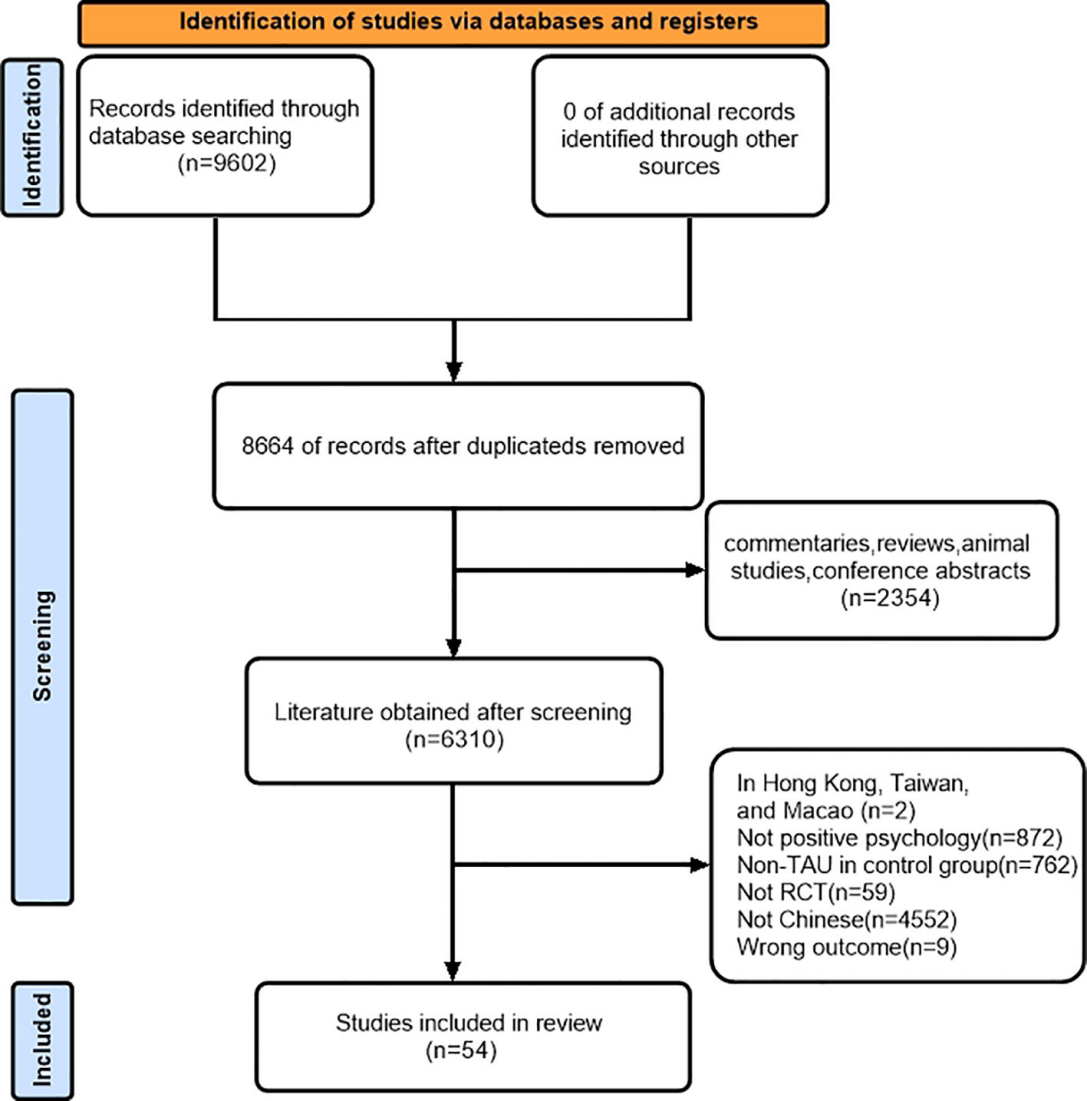
PRISMA follow diagram of the study selection process.

We conducted a comprehensive search of the database and identified 9602 studies that potentially met the criteria. After reviewing for duplicate articles, 8664 articles remained, and then 2354 articles were excluded after carefully reading the titles and abstracts. Based on the inclusion and exclusion criteria, we selected 54 articles for this systematic review and meta-analysis by reading the full text (**Figure 1**).

### Characteristics of the included studies

This meta-analysis includes 54 journal articles published from 2013 to 2024. These studies involve a total of 4,108 research subjects. The average age of the subjects in the experimental group is 40.7 ± 5.78 years, while that of the subjects in the control group is 40.48 ± 5.74 years. The intervention duration ranges from 2 to 24 weeks. All 54 RCTs compared positive psychology with TAU (such as routine care and education). Details are shown in **Table 1**.

**Table 1:** Characteristics of the included studies.

Study	Country/area	Design	Sample (IG/CG)	Mean age (IG/CG)	Experimental group	Control group	Duration/assessment time	Outcome measures
Zunqing Li (2013) (56)	China/Shandong	RCT	84 (41/43)	IG: 34.7 ± 12.3; CG: 33.8 ± 11.9	Positive psychology theory	TAU	10 weeks Once a day for 45–60 min	HEIQ, SRHMS
Feng Lin (2015) (37)	China/Jiangsu	RCT	90 (45/45)	IG: 37.53 ± 5.34; CG: 37.42 ± 5.31	Positive psychology theory	TAU	10 weeks	HEIQ, SRHMS
Liangjing Shu (2015) (40)	China/Jiangxi	RCT	72 (36/36)	IG: 33.72 ± 11.87; CG: 34.68 ± 12.21	Positive psychology theory	TAU	10 weeks	HEIQ, SRHMS
Ling Chen (2015) (41)	China/Guangdong	RCT	80 (40/40)	IG: 35.35 ± 9.28; CG: 35.13 ± 9.75	Positive psychology theory	TAU	10 weeks Once a day for 45–60 min	HEIQ
Aixia Jiao (2015) (36)	China/Shandong	RCT	98 (49/49)	IG: 39.27 ± 4.63; CG: 38.69 ± 4.52	Positive psychology theory	TAU	12 weeks	HEIQ
Chunqing Wu (2015) (76)	China/Shanghai	RCT	98 (49/49)	Total: 38.1 ± 5.1	Positive psychology theory	TAU	6 months	SDSS
Xiying Liu (2016) (49)	China/Sichuan	RCT	90 (45/45)	Total: 32.8 ± 9.8	Positive psychology theory	TAU	10 weeks	HEIQ, SRHMS
Yahan Wu (2016) (50)	China/Neimenggu	RCT	76 (38/38)	IG: 30.03 ± 3.26; CG: 30.51 ± 2.86	Positive psychology theory	TAU	10 weeks	HEIQ
Yu Fan (2016) (77)	China/Sichuan	RCT	74 (37/37)	IG: 35.09 ± 8.43; CG: 34.28 ± 7.51	Positive psychology theory	TAU	NR	SDSS, SES
Wanxia Zhao (2016) (61)	China/Shandong	RCT	82 (41/41)	IG: 37.63 ± 7.18; CG: 36.94 ± 7.06	Positive psychology theory	TAU	9 weeks	SRHMS
Junfeng Chen (2016) (85)	China/Jiangsu	RCT	98 (49/49)	IG: 36.68 ± 3.66; CG: 48.17 ± 3.59	Positive psychology theory	TAU	8 weeks	SAFE
Nina Cao (2017) (43)	China/Shanxi	RCT	100 (52/48)	IG: 43.45 ± 2.01; CG: 43.23 ± 2.33	Positive psychology theory	TAU	3 months	HEIQ, SRHMS, SDSS
Xuebo Wang (2017) (63)	China/Chongqing	RCT	48 (24/24)	IG: 42.3 ± 3.2; CG: 42.7 ± 3.3	Positive psychology theory	TAU	10 weeks	SRHMS
Yuanyuan Xu (2018) (53)	China/Jiangsu	RCT	60 (30/30)	IG: 36.16 ± 7.41; CG: 34.73 ± 7.18	Positive psychology theory	TAU	12 weeks	HEIQ, SRHMS
Yongmei Shen (2018) (52)	China/Fujian	RCT	94 (47/47)	IG: 40.32 ± 7.51; CG: 39.53 ± 6.79	Positive psychology theory	TAU	12 weeks	HEIQ, SRHMS
Jing Xu (2018) (86)	China/Beijing	RCT	98 (49/49)	IG: 37.57 ± 2.55; CG: 47.06 ± 2.48	Positive psychology theory	TAU	9 weeks	SAFE, Mo-Ca
Juan Liu (2018) (58)	China/Liaoning	RCT	104 (40/40)	IG: 41.4 ± 5.8; CG: 40.3 ± 5.6	Positive psychology theory	TAU	10 weeks	SRHMS
Jian Ni (2019) (38)	China/Shanghai	RCT	80 (40/40)	IG: 35.8 ± 11.3; CG: 34.7 ± 10.3	Positive psychology theory	TAU	10 weeks	HEIQ, SDSS, SES
Yue Feng (2019) (54)	China/Zhejiang	RCT	60 (30/30)	IG: 29.0 ± 2.8; CG: 28.9 ± 2.9	Positive psychology theory	TAU	10 weeks Once a day for 45–60 min	HEIQ, SRHMS
Xiaoxue Hu (2019) (46)	China/Jiangsu	RCT	68 (34/34)	IG: 42.18 ± 4.46; CG: 41.69 ± 4.17	Positive psychology theory	TAU	20 days	HEIQ, SRHMS
Xueling Liang (2019) (64)	China/Guangdong	RCT	96 (48/48)	IG: 41.32 ± 5.37; CG: 41.43 ± 5.41	Positive psychology theory	TAU	10 weeks Once a day for 45–60 min	SRHMS
Tingting Tao (2019) (60)	China/Henan	RCT	106 (53/53)	IG: 40.8 ± 5.7; CG: 42.5 ± 6.4	Positive psychology theory	TAU	NR	SRHMS
Guangjing Bai (2019) (78)	China/Tianjin	RCT	88 (44/44)	NR	Positive psychology theory	TAU	NR	SDSS
Jinyun Lu (2019) (79)	China/Guangdong	RCT	120 (60/60)	IG: 37.94 ± 7.06; CG: 37.61 ± 6.85	Positive psychology theory	TAU	8 weeks	SDSS, SAFE
Baofen Li (2019) (68)	China/Shandong	RCT	74 (37/37)	IG: 36.1 ± 8.1; CG: 35.9 ± 7.8	Positive psychology theory	TAU	8 weeks Once a day for 45–60 min	PANSS
Yu Wang (2019) (87)	China/Jiangxi	RCT	88 (44/44)	IG: 38.75 ± 3.66; CG: 40.17 ± 3.59	Positive psychology theory	TAU	10 weeks	Mo-CA
Juanhui He (2020) (39)	China/Hunan	RCT	122 (61/61)	IG: 44.02 ± 5.03; CG: 43.89 ± 4.93	Positive psychology theory	TAU	NR	HEIQ, SRHMS
Suli Yuan (2020) (45)	China/Zhejiang	RCT	70 (35/35)	IG: 42.42 ± 11.76; CG: 42.73 ± 12.46	Positive psychology theory	TAU	3 months	HEIQ, SDSS
							Once a day for 30–45 min	
Jing Zhou (2020) (80)	China/Jiangxi	RCT	60 (30/30)	IG: 35.56 ± 5.24; CG: 35.97 ± 5.03	Positive psychology theory	TAU	2 months	SDSS, SES
Ning Tang (2020) (81)	China/Jiangxi	RCT	92 (46/46)	IG: 72.57 ± 5.75; CG: 72.85 ± 5.84	Positive psychology theory	TAU	8 weeks	SDSS, SAFE
Yaling Yu (2020) (65)	China/Shanxi	RCT	100 (50/50)	IG: 38.46 ± 5.36; CG: 37.41 ± 5.21	Positive psychology theory	TAU	10 weeks	SRHMS
Xiuhua Gu (2021) (48)	China/Jiangsu	RCT	58 (29/29)	IG: 35.66 ± 2.65; CG: 35.59 ± 2.59	Positive psychology theory	TAU	8 weeks	HEIQ, SDSS, SES
Shugai Cao (2021) (44)	China/Henan	RCT	56 (28/28)	IG: 49.45 ± 8.28; CG: 49.03 ± 8.41	Positive psychology theory	TAU	10 weeks	HEIQ
Zongfeng Liu (2021) (67)	China/Shandong	RCT	88 (44/44)	IG: 44.35 ± 5.16; CG: 44.32 ± 5.13	Positive psychology theory	TAU	10 weeks	SRHMS, SDSS, SES
Junrui Gu (2021) (82)	China/Henan	RCT	88 (44/44)	IG: 36.33 ± 10.40; CG: 36.94 ± 10.78	Positive psychology theory	TAU	10 weeks	SDSS, SES
Jiqun Xie (2021) (57)	China/Guangdong	RCT	90 (45/45)	IG: 46.5 ± 0.2; CG: 44.5 ± 0.1	Positive psychology theory	TAU	10 weeks	SRHMS
Yanling Zhu (2021) (66)	China/Guangdong	RCT	96 (48/48)	IG: 43.22 ± 1.12; CG: 43.21 ± 1.11	Positive psychology theory	TAU	3 months	SRHMS
Xiaoqin Wu (2021) (69)	China/Fujian	RCT	94 (47/47)	IG: 44.07 ± 4.21; CG: 44.68 ± 4.03	Positive psychology theory	TAU	6 months Once a week for 30 min	PANSS
Lihong Yu (2021) (88)	China/Jiangsu	RCT	98 (49/49)	IG: 49.83 ± 4.62; CG: 38.74 ± 4.62	Positive psychology theory	TAU	6 months	Mo-CA
Zhengyuan Li (2021) (84)	China/Jiangsu	RCT	60 (30/30)	IG: 42.73 ± 2.25; CG: 43.68 ± 2.03	Positive psychology theory	TAU	10 weeks	SES
Yanfang Fang (2022) (51)	China/Guangdong	RCT	60 (30/30)	IG: 39.64 ± 5.52; CG: 39.78 ± 5.65	Positive psychology theory	TAU	8 weeks	HEIQ, SRHMS
Manhong Cai (2022) (42)	China/Guangdong	RCT	84 (42/42)	IG: 39.86 ± 4.25; CG: 39.83 ± 4.28	Positive psychology theory	TAU	2 months	HEIQ, SRHMS, SDSS, PANSS
Xiaoyan Li (2022) (47)	China/Guangdong	RCT	60 (30/30)	IG: 38.87 ± 4.16; CG: 39.04 ± 4.16	Positive psychology theory	TAU	2 months	HEIQ, SDSS
Xiaoyu Liu (2022) (62)	China/Neimenggu	RCT	120 (60/60)	IG: 38.2 ± 14.1; CG: 38.5 ± 14.7	Positive psychology theory	TAU	NR	SRHMS, SDSS, PANSS
Mengbing Fu (2022) (70)	China/Guangxi	RCT	56 (28/28)	IG: 45.31 ± 7.48; CG: 45.27 ± 7.50	Positive psychology theory	TAU	3 months	PANSS
Jinping Wang (2022) (71)	China/Jiangsu	RCT	60 (30/30)	IG: 41.96 ± 3.86; CG: 41.36 ± 3.67	Positive psychology theory	TAU	3 months	PANSS
Cuilu Shi (2022) (89)	China/Hebei	RCT	80 (40/40)	IG: 38.69 ± 3.64; CG: 38.68 ± 3.65	Positive psychology theory	TAU	8 weeks	Mo-CA
Yuwen Zhang (2023) (55)	China/Jiangsu	RCT	92 (46/46)	IG: 45.02 ± 1.15; CG: 44.15 ± 1.10	Positive psychology theory	TAU	8 weeks Once every 2 weeks	HEIQ, SRHMS
Xuemei Li (2023) (72)	China/Fujian	RCT	74 (37/37)	IG: 38.27 ± 9.37; CG: 38.07 ± 10.22	Positive psychology theory	TAU	3 months	PANSS
Lingli Xu (2023) (73)	China/Jiangsu	RCT	78 (39/39)	IG: 48.37 ± 3.54; CG: 48.41 ± 3.62	Positive psychology theory	TAU	NR	PANSS
Huijuan Xing (2023) (74)	China/Neimenggu	RCT	62 (31/31)	IG: 35.95 ± 5.54; CG: 35.32 ± 5.12	Positive psychology theory	TAU	3 months	PANSS
Mengzhi Ma (2023) (59)	China/Xinjiang	RCT	90 (45/45)	IG: 48.97 ± 3.58; CG: 49.18 ± 3.64	Positive psychology theory	TAU	3 months	SRHMS
Yanlan Wu (2024) (83)	China/Jiangxi	RCT	92 (46/46)	IG: 39.63 ± 3.30; CG: 39.58 ± 3.24	Positive psychology theory	TAU	3 months 45 min twice a week	SDSS
Yanyan Fang (2024) (75)	China/Shandong	RCT	84 (42/42)	IG: 50.02 ± 10.43; CG: 50.23 ± 10.25	Positive psychology theory	TAU	2 months Three hours three times a week	PANSS

RCT, randomized controlled trial; IG, intervention groups; CG, control group; NR, not reported; TAU, treatment as usual; SRHMS, Self-rated Health Measurement Scale; HEIQ, Health Education Impact Questionnaire; PANSS, Positive and Negative Syndrome Scale; SDSS, Social Disability Screening Schedule; SES, Self-Esteem Scale; SAFE, Social Adaptability Scale; Mo-CA, Montreal Cognitive Assessment.

A total of 21 articles used the Health Education Impact Questionnaire (HEIQ) to assess wellbeing (36–56), 24 articles used the Self-rated Health Measurement Scale (SRHMS) to assess mental health (37, 39, 40, 42, 43, 46, 49, 51–67), 10 articles used the Positive and Negative Syndrome Scale (PANSS) to assess positive and negative symptoms of schizophrenia (42, 62, 68–75), 16 articles used the Social Disability Screening Schedule (SDSS) scale to assess social function (38, 42, 43, 45, 47, 48, 62, 67, 76–83), 7 articles used the Self-Esteem Scale (SES) scale to assess self-esteem (38, 48, 67, 77, 80, 82, 84), 4 articles used the Social Adaptability Scale (SAFE) scale to assess social adaptability (79, 81, 85, 86), and 4 articles used the Montreal Cognitive Assessment (Mo-CA) scale to assess cognitive function (86–89).

### Risk of bias in the included studies

**Figure 2:**
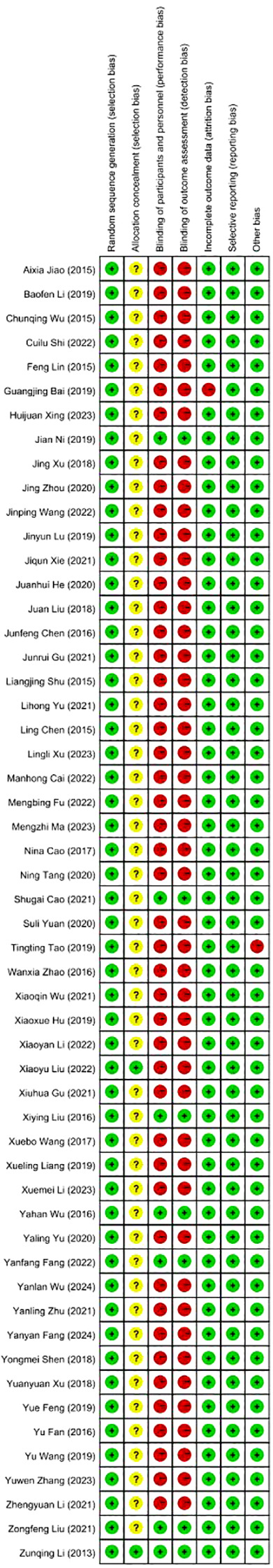
Summary of risk of bias for included studies.

**Figure 3:**
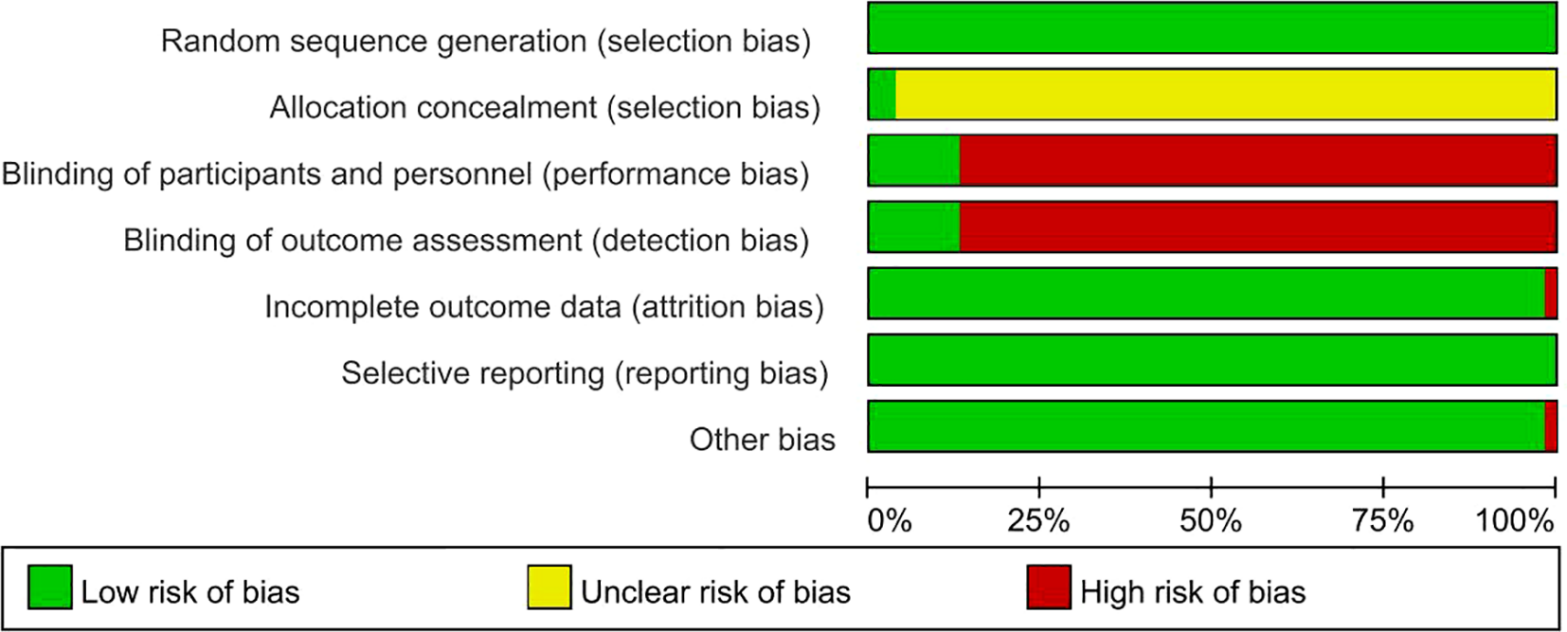
Bias risk proportion graph for included studies.

The quality of the included literature was assessed; 7 articles were classified as having a low risk of bias, 1 article scored 7 points, and the remaining 47 articles were all categorized as having a moderate risk of bias (**Figure 2**). A graph of the proportion of bias risk in the included studies is presented (**Figure 3**). The most significant risks of the included literature are the lack of randomization details and insufficient blinding methods. Specifically, 96.3% of the literature lack randomization details, and 87.1% have insufficient blinding methods.

### Effect on mental health

**Figure 4:**
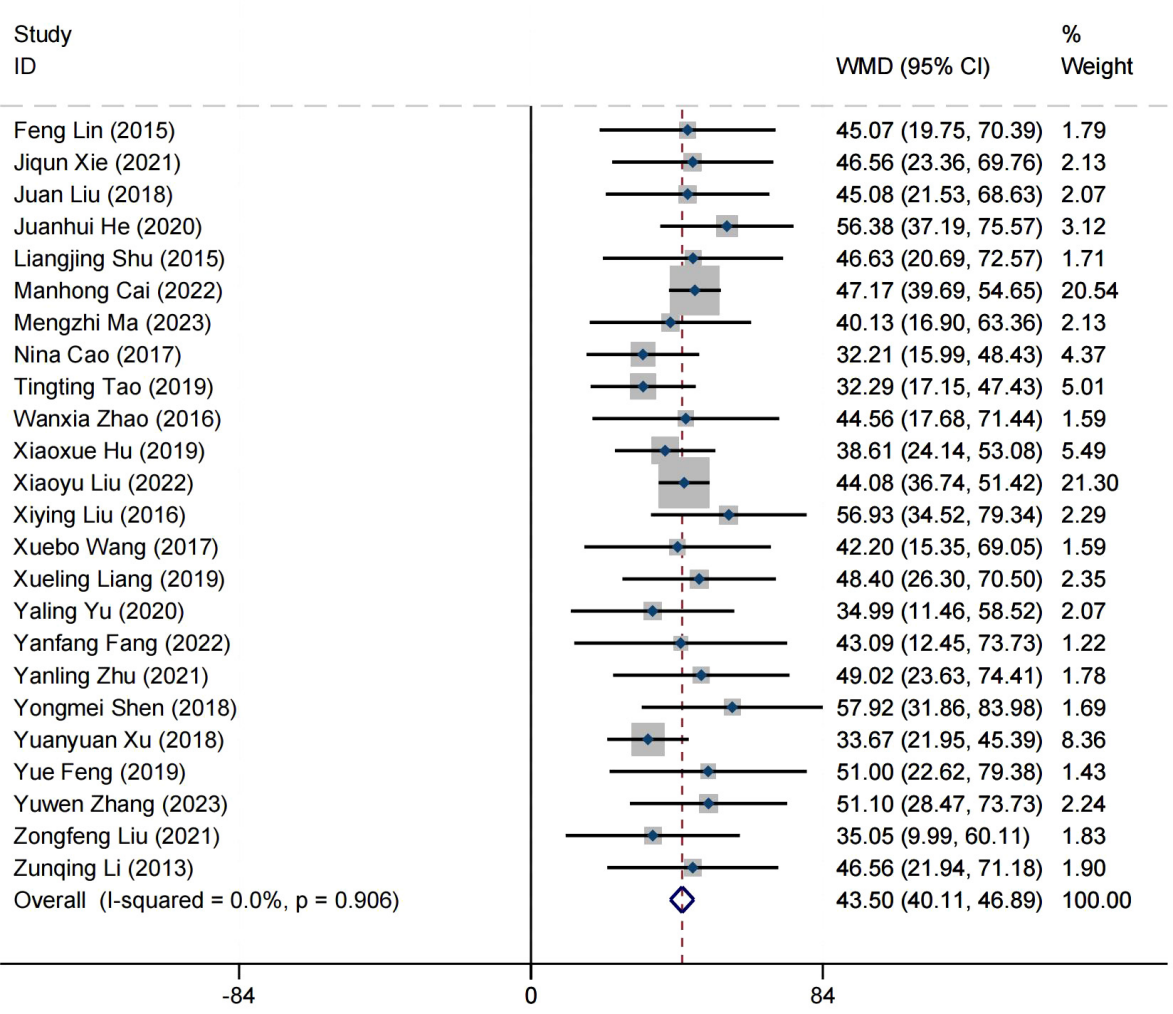
Forest plot of the effects of positive psychology on mental health.

**Figure 5:**
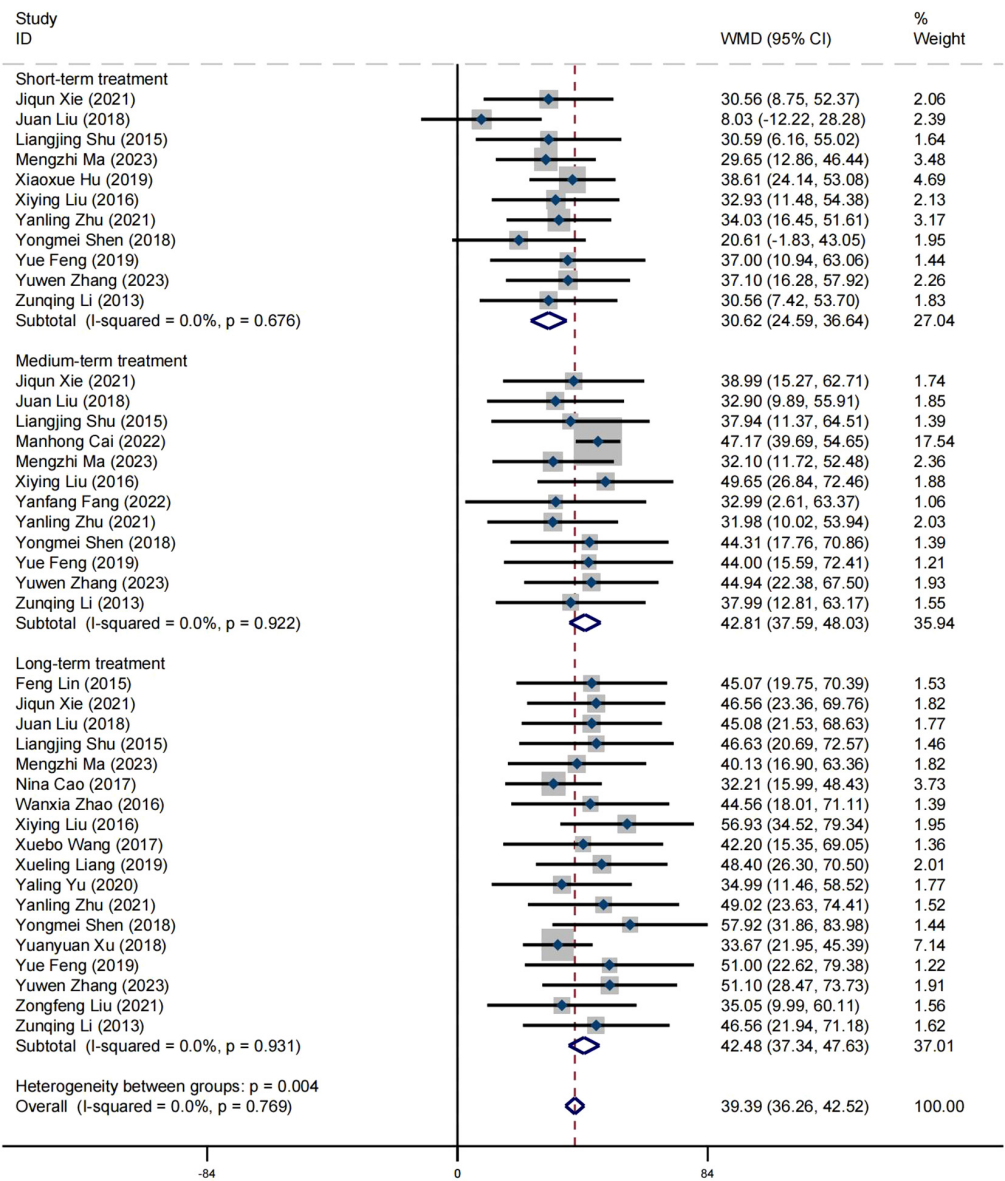
Forest plot of mental health in subgroup analyses.

A total of 23 articles reported on the impact of positive psychology theory on the mental health of patients with schizophrenia, including a total of 2,090 patients, with 1,047 in the experimental group and 1,043 in the control group. The results show that intervention with positive psychology theory can significantly improve the mental health of patients with schizophrenia, and it is markedly superior to the control group (MD = 43.50, 95% CI = 40.11 to 46.89, *p* < 0.001, *I* ^2^ = 0%) (**Figure 4**). Eleven studies reported the impact of short-term positive psychology theory intervention on the mental health of patients with schizophrenia, showing a significant increase in mental health (MD = 30.62, 95% CI = 24.59 to 36.64, *p* < 0.001). Twelve studies reported the impact of medium-term positive psychology theory intervention on the mental health of patients with schizophrenia, showing a significant increase in mental health (MD = 42.81, 95% CI = 37.59 to 48.03, *p* < 0.001). Eighteen studies reported the impact of long-term positive psychology theory intervention on the mental health of patients with schizophrenia, showing a significant increase in mental health (MD = 42.48, 95% CI = 37.34 to 47.63, *p* < 0.001). The differences among the three groups were statistically significant (*p* = 0.004), indicating that long-term and medium-term interventions with positive psychology theory are significantly better than short-term interventions for the mental health. However, there was no statistical significance between the long-term and medium-term treatment groups (*p* = 0.93 > 0.05) (**Figure 5**). According to the GRADE evidence grading assessment, the certainty level of mental health was rated as low quality, with downgrades due to the presence of bias risk and inconsistency. Details are found in **Supplementary Material S2**.

### Effect on wellbeing

**Figure 6:**
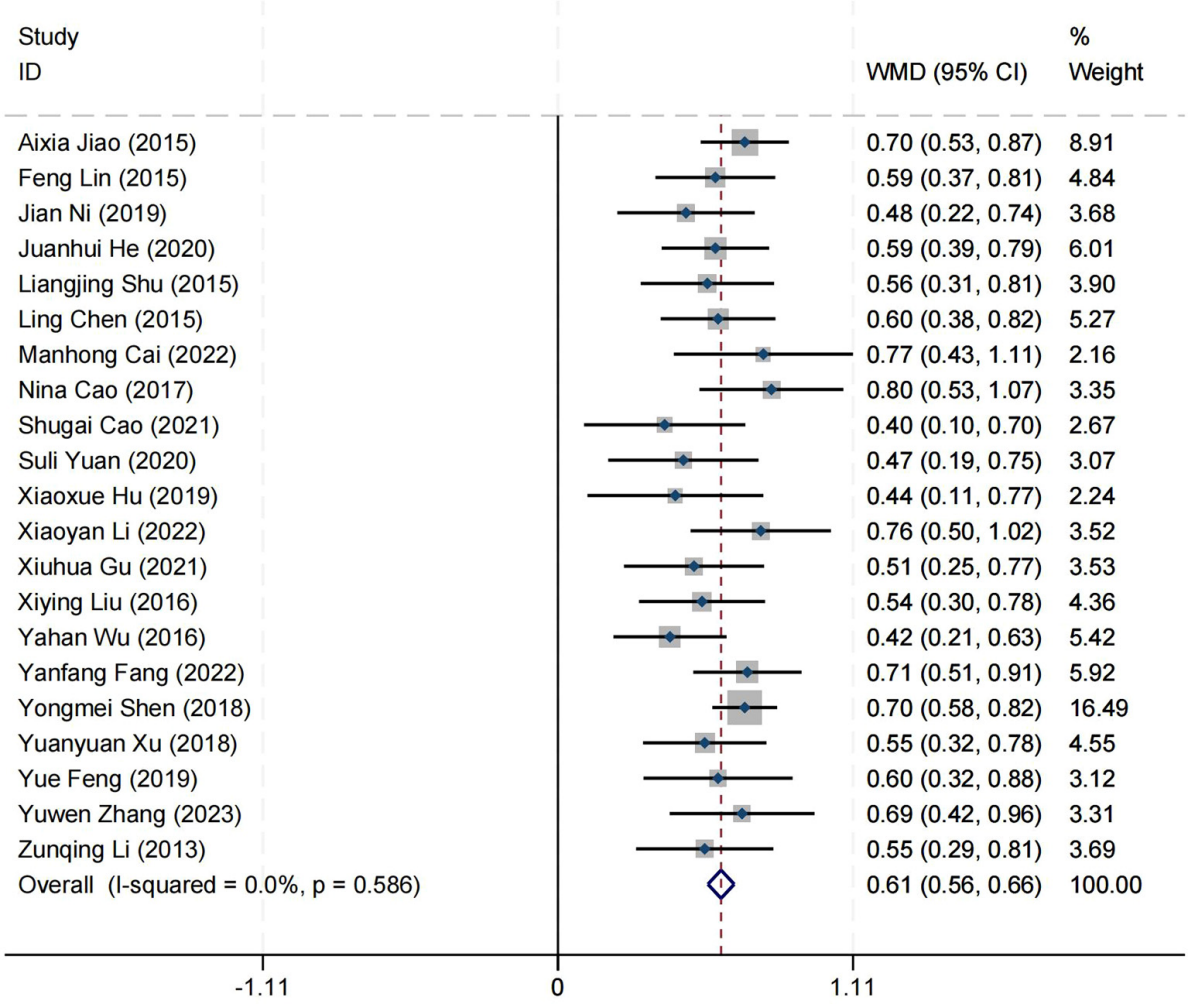
Forest plot of the effects of positive psychology on wellbeing.

**Figure 7:**
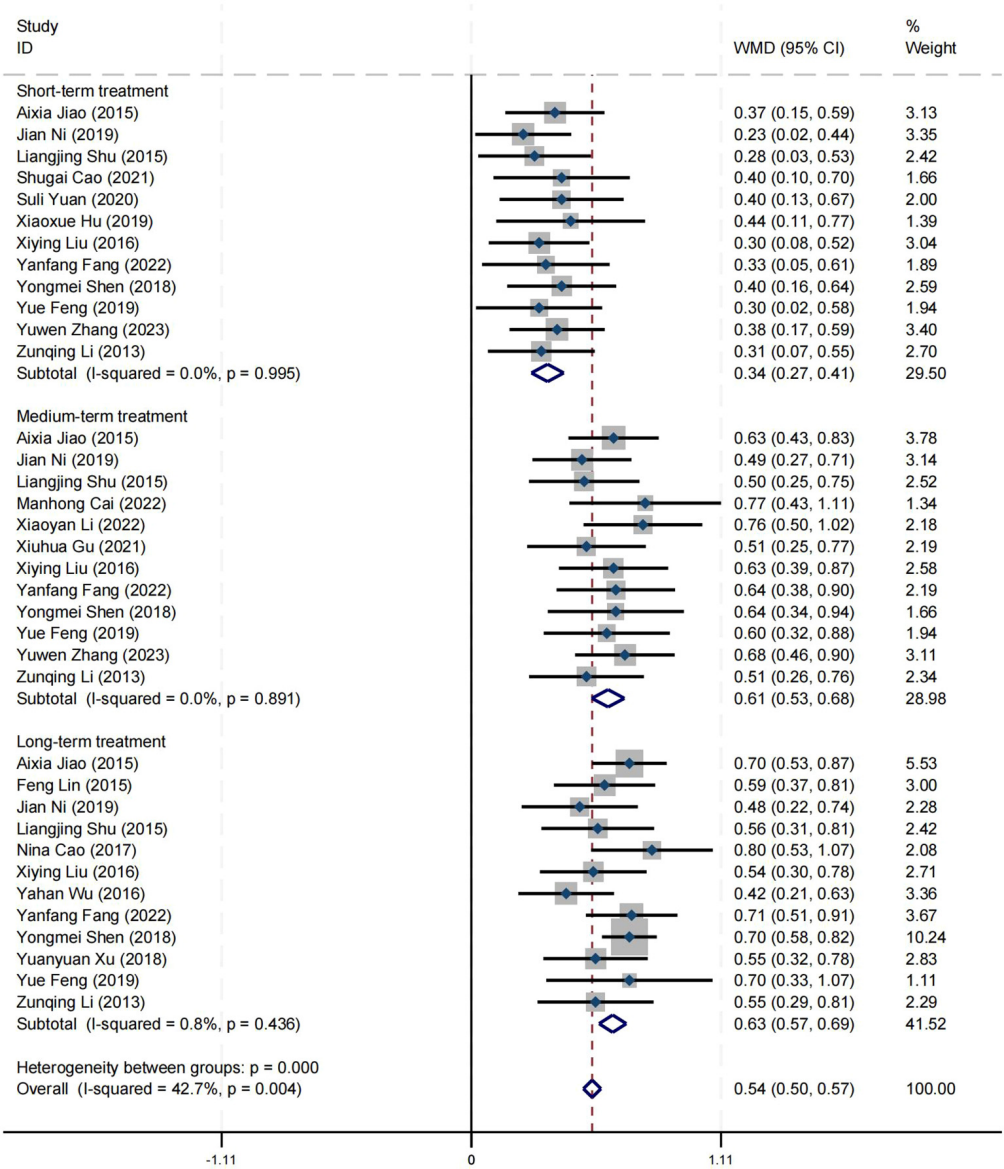
Forest plot of wellbeing in subgroup analyses.

Twenty-one articles reported on the impact of positive psychology theory on the wellbeing of patients with schizophrenia, including a total of 1,650 patients, with 827 in the experimental group and 823 in the control group. The results show that the intervention of positive psychology theory can significantly improve the wellbeing of patients with schizophrenia, and it is obviously better than the control group (MD = 0.61, 95% CI = 0.56 to 0.66, *p* < 0.001, *I* ^2^ = 0%) (**Figure 6**). Twelve studies reported the impact of short-term positive psychology theory intervention on the wellbeing of patients with schizophrenia, showing a significant increase in scores (MD = 0.34, 95% CI = 0.27 to 0.41, *p* < 0.001). Twelve studies reported the impact of medium-term positive psychology theory intervention on the wellbeing of patients with schizophrenia, showing a significant increase in scores (MD = 0.61, 95% CI = 0.53 to 0.68, *p* < 0.001). Twelve studies reported the impact of long-term positive psychology theory intervention on the wellbeing of patients with schizophrenia, showing a significant increase in scores (MD = 0.63, 95% CI = 0.57 to 0.69, *p* < 0.001). The differences among the three groups were statistically significant (*p* < 0.001), indicating that long-term and medium-term interventions with positive psychology theory are significantly better than short-term intervention for the wellbeing of patients with schizophrenia. However, there was no statistical significance between the long-term and medium-term treatment groups (*p* = 0.78 > 0.05) (**Figure 7**). According to the GRADE evidence grading assessment, the certainty level of wellbeing was rated as moderate quality, with downgrades due to the presence of bias risk. Details are found in **Supplementary Material S2**.

### Effect on positive symptom

**Figure 8:**
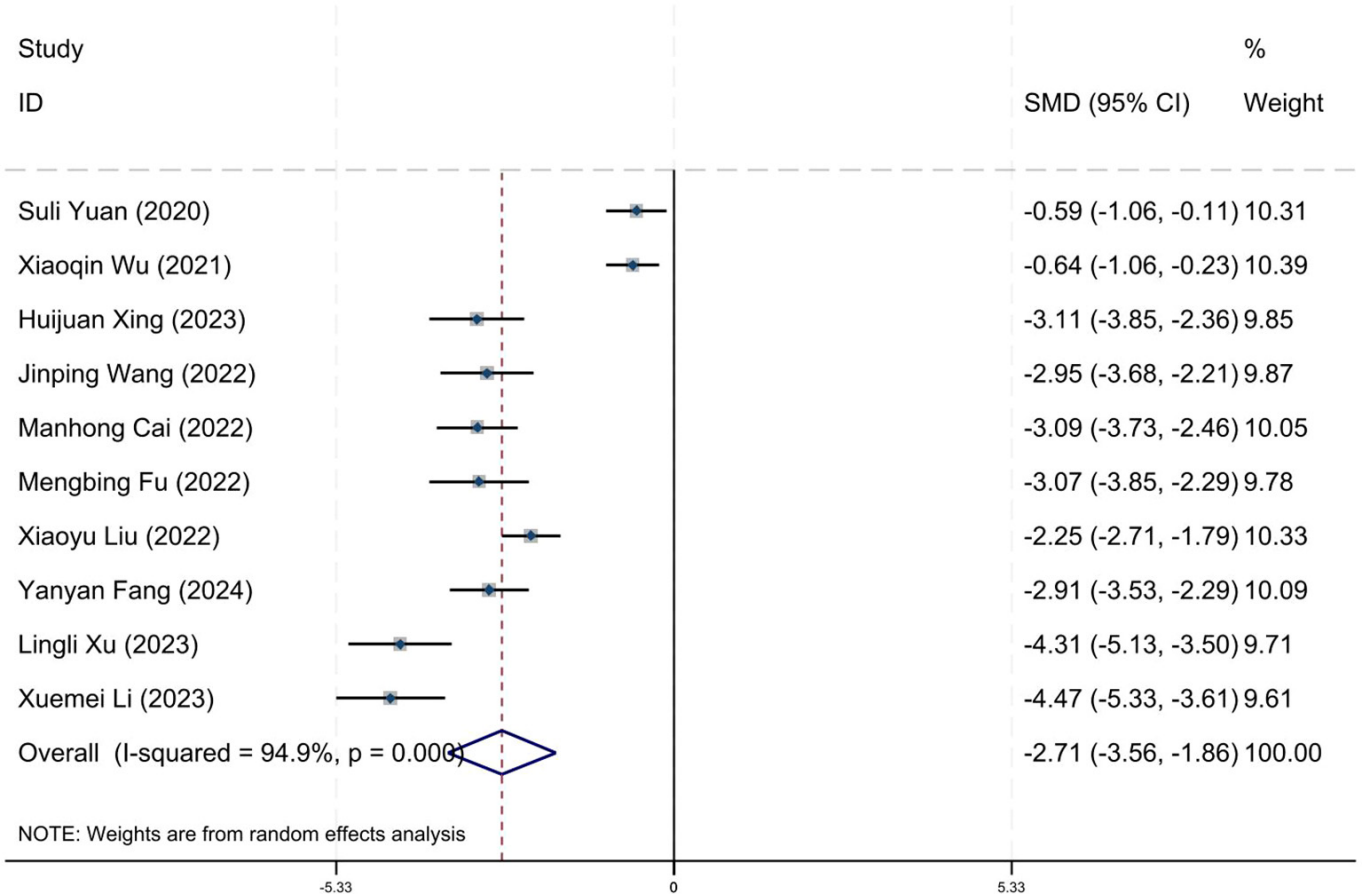
Forest plot of the effects of positive psychology on positive symptom.

**Figure 9:**
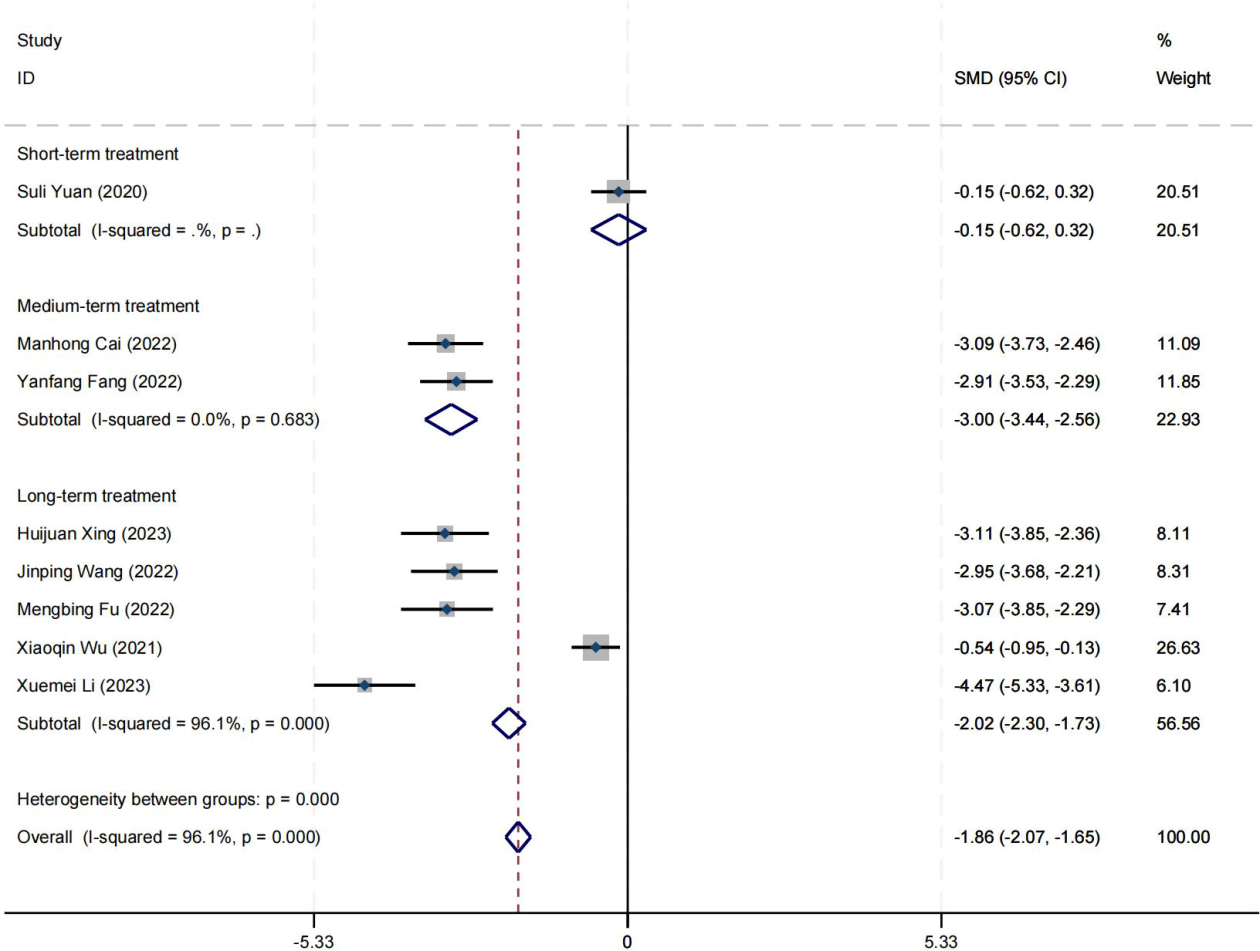
Forest plot of positive symptom in subgroup analyses.

Ten research studies have reported the impact of positive psychology theory on the recovery of positive symptoms in schizophrenia. The results show that positive psychology theory can significantly improve the positive symptoms of schizophrenia and is superior to TAU (SMD = −2.68, 95% CI = −3.53 to −1.84, *p* < 0.001, *I* ^2^ = 95%) (**Figure 8**). The results of sensitivity analysis showed values of SMDs ranging from −2.68 (95% CI = −3.53 to −1.84) to −3.19 (95% CI = −3.70 to −2.98), with *I*² ranging from 95% to 77% (**Supplementary Material S4**). The results of the sensitivity analysis were stable. One study reported the impact of short-term positive psychology theory intervention on the positive symptoms of schizophrenia, with results showing no significant effect (SMD = −0.15, 95% CI = −0.62 to 0.32, *p* > 0.05). Two studies reported the impact of medium-term positive psychology theory intervention on the positive symptoms of schizophrenia, showing a significant improvement in the positive symptoms of schizophrenia (SMD = −2.97, 95% CI = −3.42 to −2.53, *p* < 0.001). Six studies reported the impact of long-term positive psychology theory intervention on the positive symptoms of schizophrenia, showing a significant improvement in the positive symptoms of schizophrenia (SMD = −2.77, 95% CI = −4.27 to −1.27, *p* < 0.001). The differences among the three groups were statistically significant (*p* < 0.001), indicating that long-term and medium-term interventions with positive psychology theory are significantly better than short-term interventions for the positive symptoms of schizophrenia. However, there was no statistical significance between the long-term and medium-term treatment groups (*p* = 0.80 > 0.05) (**Figure 9**). According to the GRADE evidence grading assessment, the certainty level of the positive symptom was rated as low quality, with the downgrade due to the presence of bias risk and publication bias. Details are found in **Supplementary Material S2**.

### Effect on negative symptom

**Figure 10:**
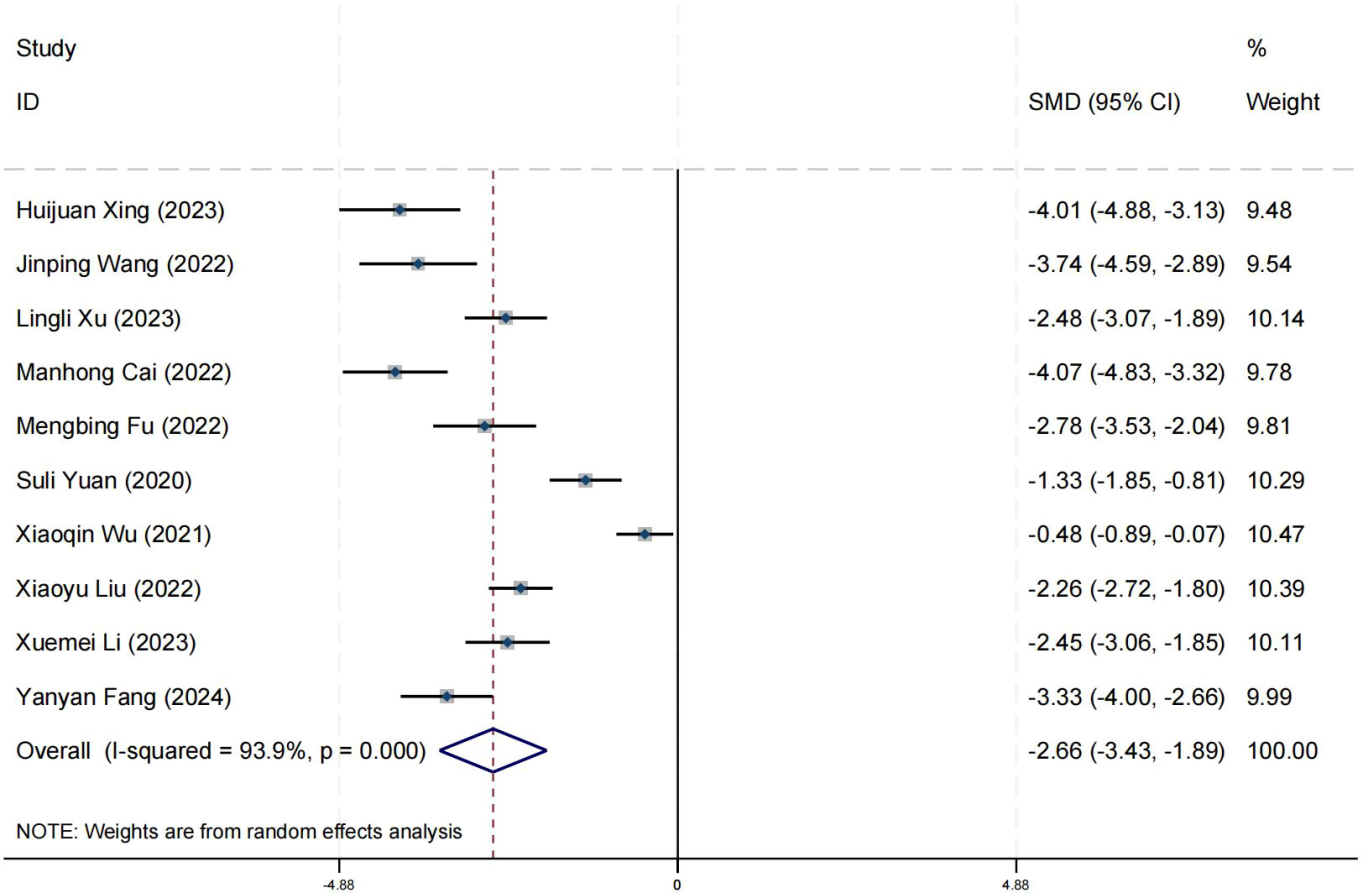
Forest plot of the effects of positive psychology on negative symptom.

**Figure 11:**
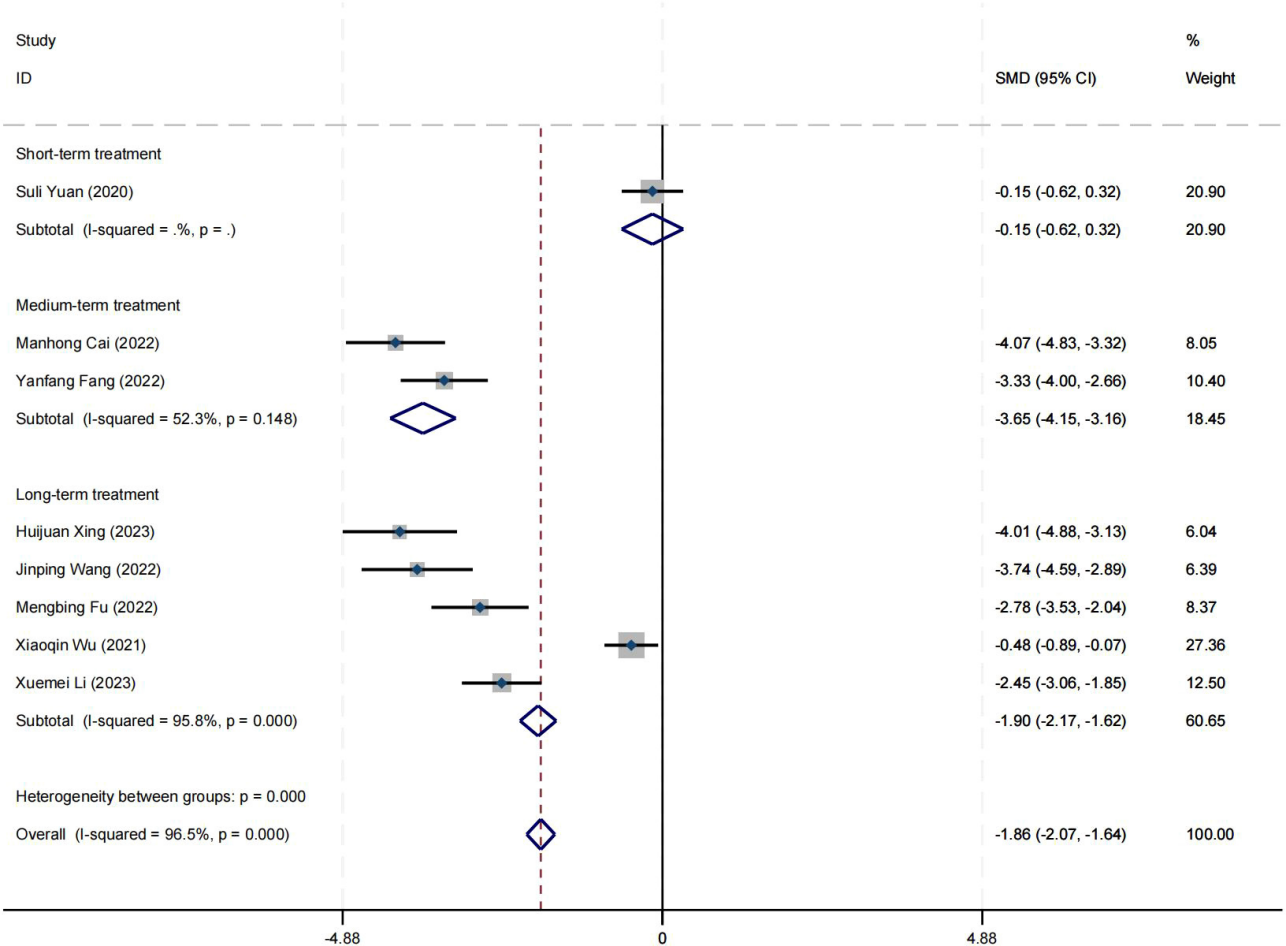
Forest plot of negative symptom in subgroup analyses.

Ten research studies have reported the impact of positive psychology theory on the recovery of negative symptoms in schizophrenia. The results show that positive psychology theory can significantly improve the negative symptoms of schizophrenia and is superior to TAU (SMD = −2.63, 95% CI = −3.40 to −1.87, *p* < 0.001, *I* ^2^ = 94%) (**Figure 10**). The results of sensitivity analysis showed values of SMDs ranging from −2.63 (95% CI = −3.40 to −1.87) to −3.06 (95% CI = −3.56 to −2.56), with *I*² ranging from 94% to 78% (**Supplementary Material S5**). The results of the sensitivity analysis were stable. One study reported the impact of short-term positive psychology theory intervention on the negative symptoms of schizophrenia, with results showing no significant effect (SMD = −0.15, 95% CI = −0.62 to 0.32). Two studies reported the impact of medium-term positive psychology theory intervention on the negative symptoms of schizophrenia, showing a significant improvement in the negative symptoms of schizophrenia (SMD = −3.64, 95% CI = −4.37 to −2.92, *p* < 0.001). Five studies reported the impact of long-term positive psychology theory intervention on the negative symptoms of schizophrenia, showing a significant improvement in the negative symptoms of schizophrenia (SMD = −2.63, 95% CI = −4.04 to −1.22, *p* < 0.003). The differences among the three groups were statistically significant (*p* < 0.001), indicating that long-term and medium-term interventions with positive psychology theory are significantly better than short-term interventions for the negative symptoms of schizophrenia. However, there was no statistical significance between the long-term and medium-term treatment groups (*p* = 0.21 > 0.05) (**Figure 11**). According to the GRADE evidence grading assessment, the certainty level of the negative symptom was rated as low quality, with the downgrade due to the presence of bias risk and publication bias. Details are found in **Supplementary Material S2**.

### Effect on PANSS-total

**Figure 12:**
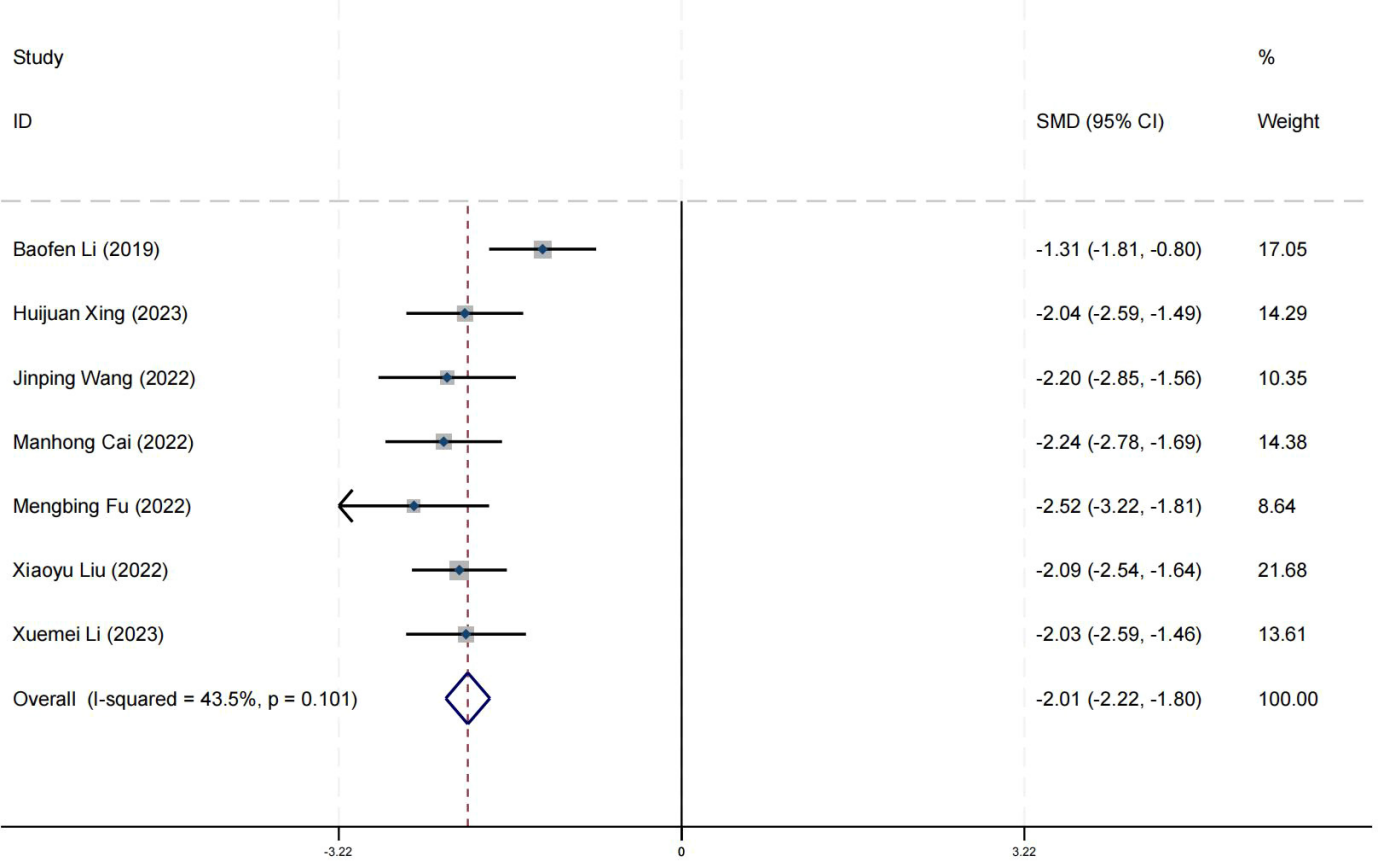
Forest plot of the effects of positive psychology on PANSS-Total.

Seven research studies have reported on the improvement of schizophrenia symptoms through positive psychology theory. The results show that positive psychology theory can significantly reduce PANSS-Total, and is superior to TAU (SMD = −1.99, 95% CI = −2.20 to −1.78, *p* < 0.001, *I*² = 42%) (**Figure 12**). According to the GRADE evidence grading assessment, the certainty level of PANSS-Total was rated as low quality, with the downgrade due to the presence of bias and imprecision risks. Details are found in **Supplementary Material S2**.

### Effect on social function

**Figure 13:**
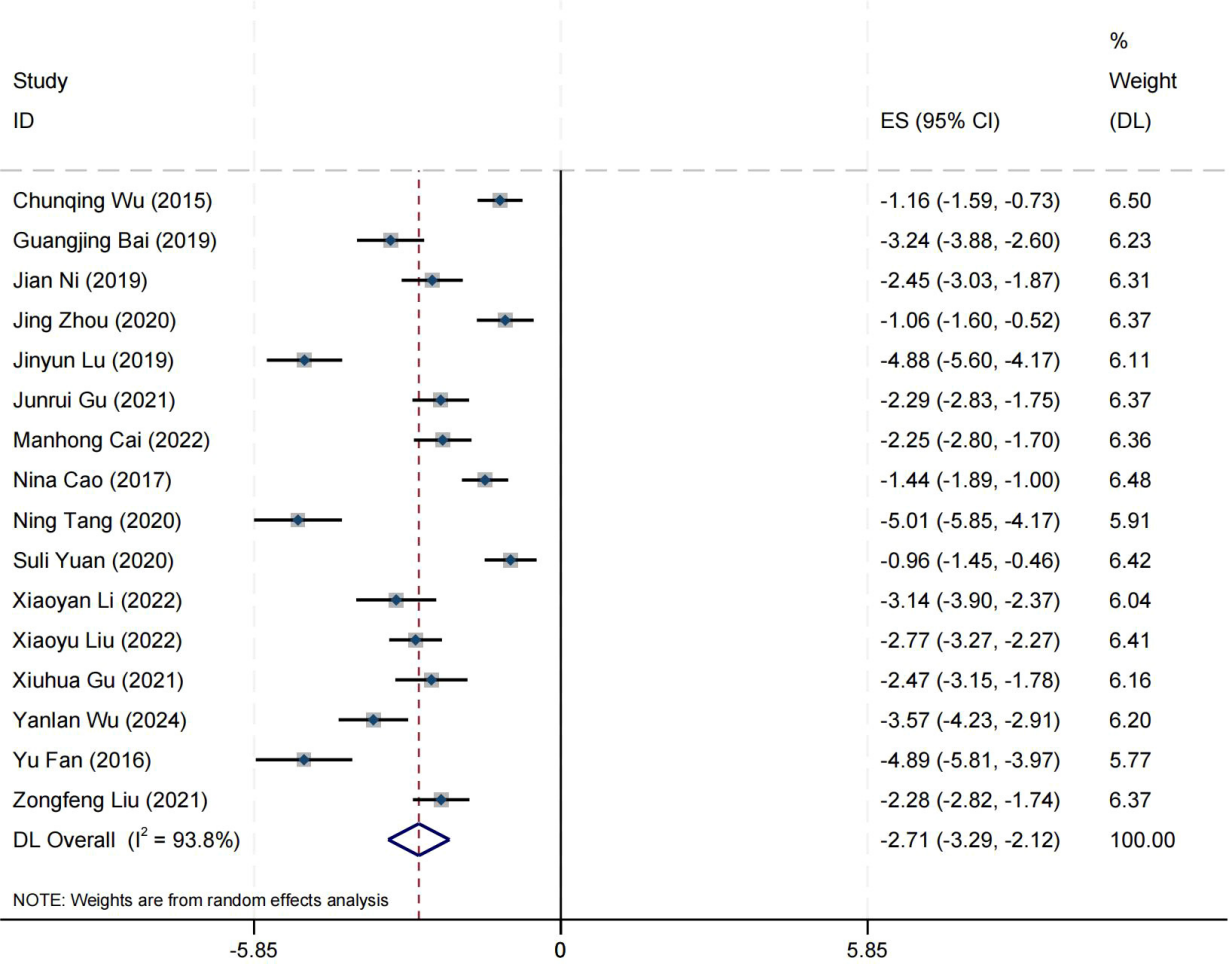
Forest plot of the effects of positive psychology on social function.

**Figure 14:**
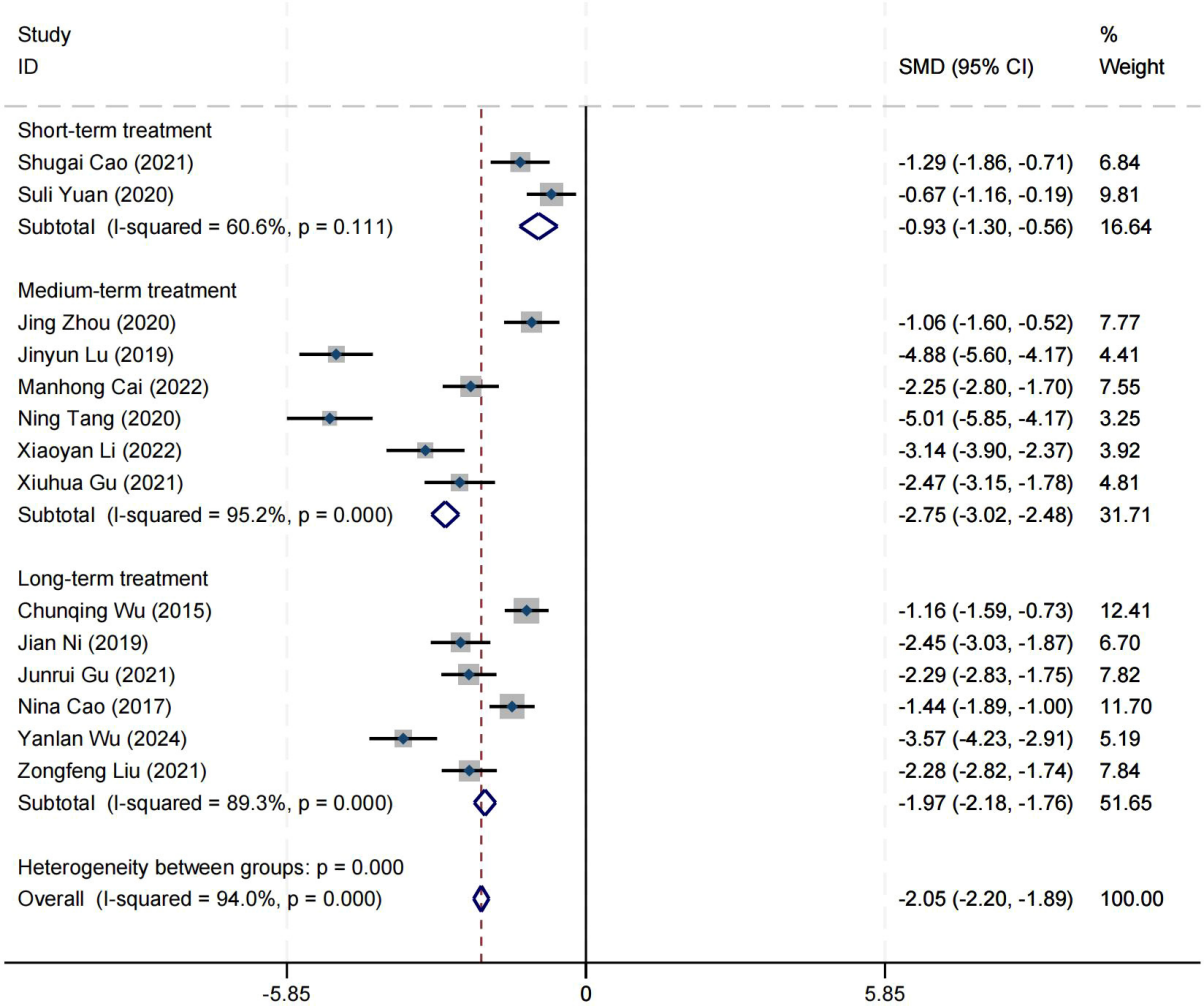
Forest plot of social function in subgroup analyses.

Sixteen research studies have reported on the impact of positive psychology theory on the recovery of social function in patients with schizophrenia. The results indicate that positive psychology theory can significantly improve the social function of patients with schizophrenia and is superior to TAU (MD = −2.68, 95% CI = −3.26 to −2.10, *p* < 0.001, *I* ^2^ = 94%) (**Figure 13**). The results of sensitivity analysis showed values of SMDs ranging from −2.68 (95% CI = −3.26 to −2.10) to −2.63 (95% CI = −3.19 to −2.08), with *I*² ranging from 94% to 92% (**Supplementary Material S6**). The results of the sensitivity analysis were stable. Two studies reported the impact of short-term positive psychology theory intervention on social function, showing a significant improvement in the social function of patients with schizophrenia (SMD = −0.95, 95% CI = −1.53 to 0.36, *p* = 0.002). Six studies reported the impact of medium-term positive psychology theory intervention on social functioning, showing a significant improvement in the social function of patients with schizophrenia (SMD = −3.09, 95% CI = −4.32 to −1.85, *p* < 0.001). Six studies reported the impact of long-term positive psychology theory intervention on social function, showing a significant improvement in the social function of patients with schizophrenia (SMD = −2.15, 95% CI = −2.80 to −1.51, *p* < 0.001). The differences among the three groups were statistically significant (*p* < 0.001), indicating that long-term and medium-term interventions with positive psychology theory are significantly better than short-term intervention for the social function of patients with schizophrenia. However, there was no statistical significance between the long-term and medium-term treatment groups (*p* = 0.19 > 0.05) (**Figure 14**). According to the GRADE evidence grading assessment, the certainty level of the social function was rated as low quality, with the downgrade due to the presence of bias risk, inconsistency, and publication bias. Details can be found in **Supplementary Material S2**.

### Effect on self-esteem

**Figure 15:**
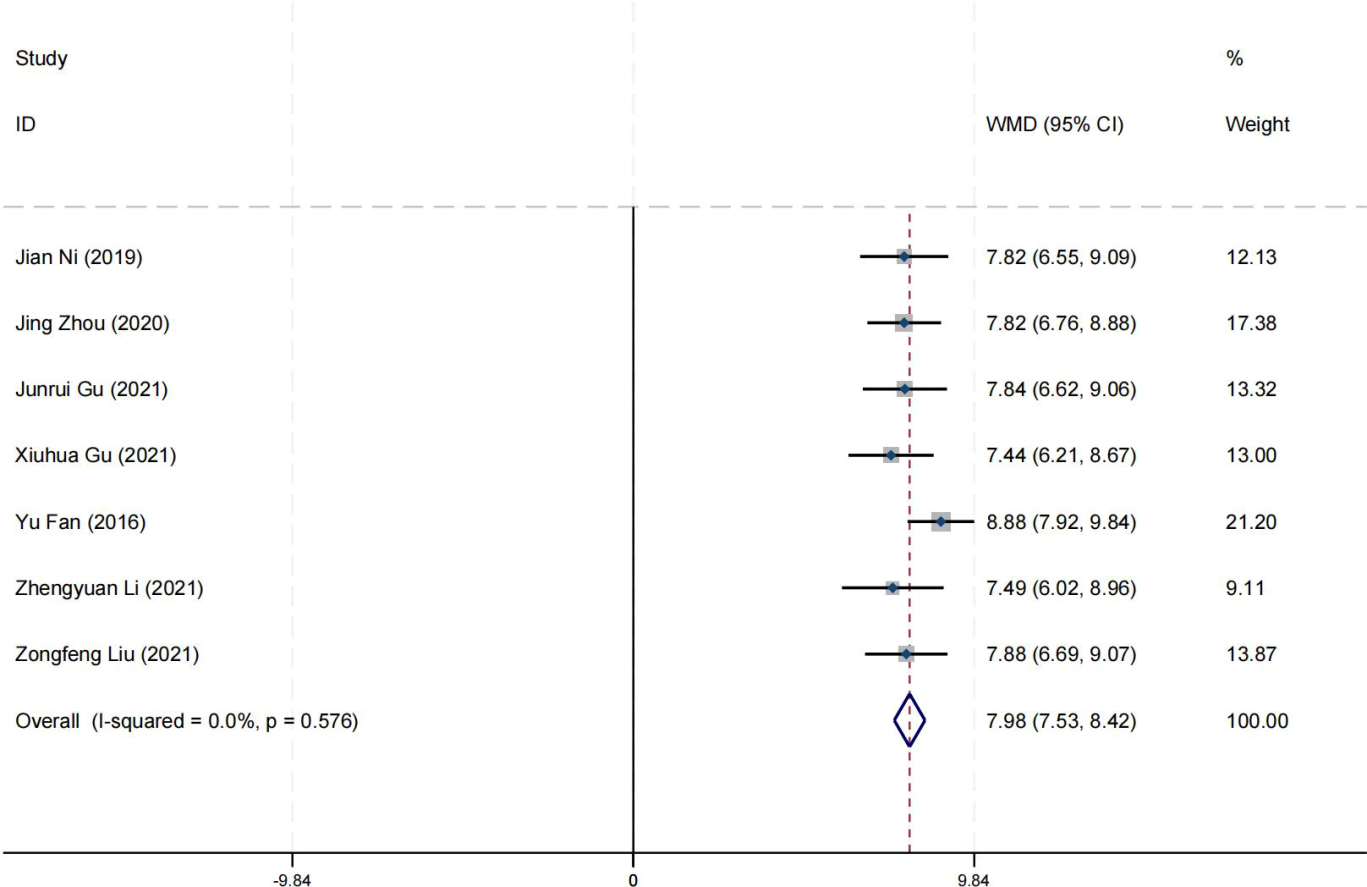
Forest plot of the effects of positive psychology on self-esteem.

Seven research studies have reported on the impact of positive psychology theory on the self-esteem of patients with schizophrenia. The results show that positive psychology theory can significantly improve the self-esteem of patients with schizophrenia and is superior to TAU (MD = 7.98, 95% CI = −7.53 to 8.42, *p* < 0.001, *I*² = 0%) (**Figure 15**). According to the GRADE evidence grading assessment, the certainty level of the self-esteem was rated as low quality, with the downgrade due to the presence of bias and imprecision risks. Details are found in **Supplementary Material S2**.

### Effect on social adaptability

**Figure 16:**
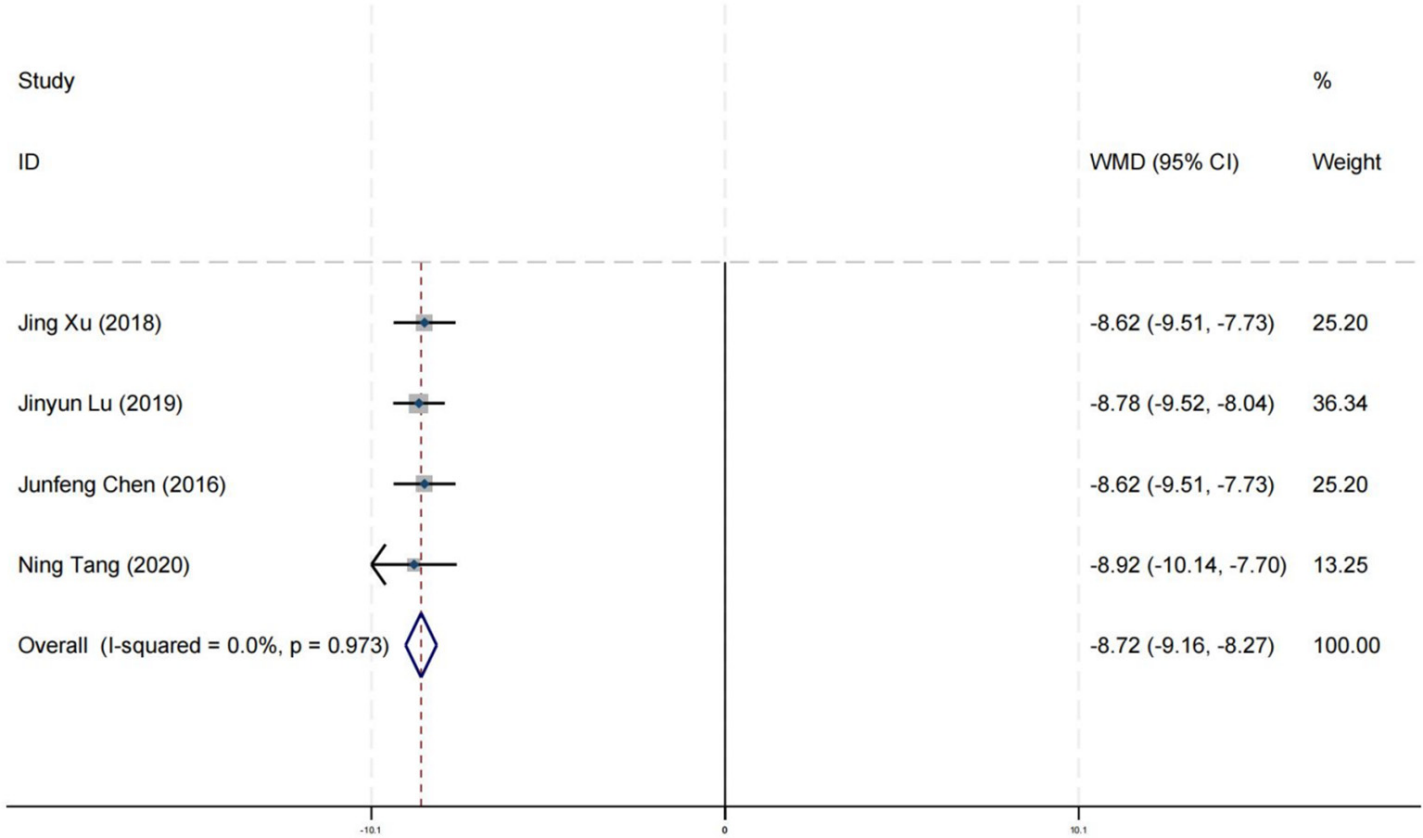
Forest plot of the effects of positive psychology on social adaptability.

Four research studies have reported on the impact of positive psychology theory on the social adaptability of patients with schizophrenia. The results show that positive psychology theory can significantly improve the social adaptability of patients with schizophrenia and is superior to TAU (MD = −8.72, 95% CI = −9.16 to −8.27, *p* < 0.001, *I^2^* = 0%) (**Figure 16**). According to the GRADE evidence grading assessment, the certainty level of the social adaptability was rated as low quality, with the downgrade due to the presence of bias and imprecision risks. Details are found in **Supplementary Material S2**.

### Effect on cognitive function

**Figure 17:**
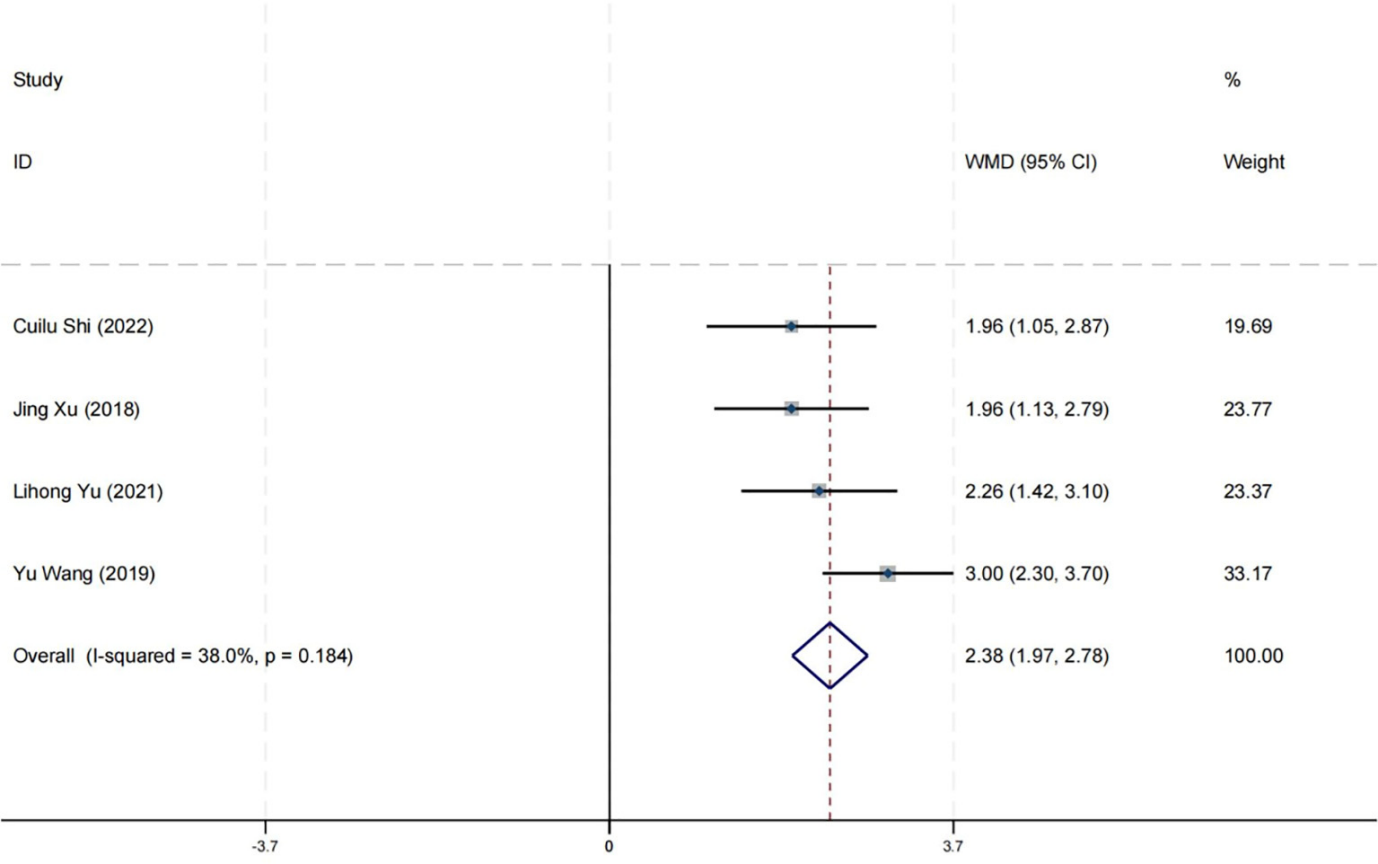
Forest plot of the effects of positive psychology on cognitive function.

Four research studies have reported on the impact of positive psychology theory on the cognitive function of patients with schizophrenia. The results show that positive psychology theory can significantly improve the cognitive function of patients with schizophrenia and is superior to TAU (MD = 2.38, 95% CI = 1.97 to 2.78, *p* < 0.001, *I* ^2^ = 38%) (**Figure 17**). According to the GRADE evidence grading assessment, the certainty level of the cognitive function was rated as low quality, with the downgrade due to the presence of bias risk and imprecision. Details are found in **Supplementary Material S2**.

### Publication bias

This study included 54 articles and conducted a publication bias test on various outcome indicators (90). The funnel plots of mental health and wellbeing were symmetrical (**Supplementary Material S10**), and the Egger’s test was conducted with *p* > 0.05. The funnel plots of positive symptoms, negative symptoms, and social function showed asymmetry, and the Egger’s test was conducted with *p* < 0.05 (**Supplementary Material S10**). The results of the trim-and-fill method were consistent with the original results (35). Details can be found in **Supplementary Materials S7**, **S8**, and **S9**.

## Discussion

Schizophrenia is a long-term and multifaceted mental disorder, with core symptoms including disorganized thinking, hallucinations, delusions, affective flattening, social withdrawal, and cognitive impairments (13). Patients may experience auditory or visual illusions of things that do not exist, hold beliefs that are not in line with reality, and face challenges in expressing emotions and forming social connections (91). These difficulties can significantly disrupt daily life and place stress on families (92–94). With the trend in contemporary healthcare, positive psychology has gradually attracted widespread attention due to its safety, efficacy, few side effects, and patient acceptance, and it is being increasingly applied in the clinical treatment of various mental disorders (21, 95). In this meta-analysis, we have conducted a comprehensive review of the application and efficacy of positive psychology in the treatment of patients with schizophrenia in China. To our knowledge, this is the first and most extensive meta-analysis of intervention effects based on positive psychology theory for patients with schizophrenia in China. The analysis shows that positive psychology can effectively enhance the wellbeing, mental health, self-esteem, cognitive function, social function, and social adaptability of patients with schizophrenia and can significantly improve their positive and negative symptoms, as well as depressive symptoms. Although psychological intervention research for patients with schizophrenia is quite common (96, 97), comprehensive meta-analyses conducted by foreign researchers often exclude Chinese studies because they are published in non-English languages. In addition, previous meta-analyses published abroad have been unclear due to high heterogeneity and inconsistent use of measurement scales with small sample sizes, and the conclusions on enhancing the wellbeing of patients with schizophrenia are not clear, and the efficacy on positive symptoms is not significant (28). The intervention measures included in the studies were not completely uniform, and the intervention time was also short. Since positive psychology intervention aims to change the way individuals face life challenges, a longer intervention time is needed to further clarify its clinical significance. In this study, we expanded the sample size, unified the intervention methods, and conducted subgroup analyses on four indicators: wellbeing, mental health, positive and negative symptoms of schizophrenia, and social function at different time periods. The analysis results show that the effects of long-term and medium-term positive psychology interventions are significantly better than those of short-term interventions, but there is no significant difference between medium-term and long-term interventions. This may be due to the particularity of schizophrenia and the inconsistency in the measurement scales and researcher scoring standards used. In the analysis of social function, positive and negative symptoms of schizophrenia, the SDSS, and the PANSS were used. We adopted strict inclusion criteria, a random-effects model, and moderator analyses (including meta-regression and subgroup analyses) to control and reduce heterogeneity. However, despite these measures, the moderator analysis still failed to reduce the *I*² value to 75% or lower. The heterogeneity remained relatively high. We attempted to explore the sources of heterogeneity by reviewing the literature and communicating with the authors. We found that individual differences among patients with schizophrenia, different therapist intervention methods, the limited number of studies included in this indicator, and the inconsistency of years and regions led to specific modifications of the questionnaires used. For example, in some studies measuring the negative symptoms of schizophrenia using the SDSS and PANSS, the effect sizes were significantly smaller (45, 69). Considering these factors, although our analysis indicates that positive psychological interventions have a certain impact on the social function and the positive and negative symptoms of patients with schizophrenia, the high heterogeneity should be taken into account when interpreting the conclusions. The same caution should be applied when applying these research findings to clinical practice or further studies, fully considering this uncertainty.

In this study, we conducted a rigorous assessment of publication bias. Through visual analysis of funnel plots and Egger’s test, we initially determined the presence of publication bias. Subsequently, we further analyzed using the trim-and-fill method, and the results showed that the research outcomes of social functioning, positive symptoms, and negative symptoms exhibited significant publication bias (*p* < 0.05). In the field of psychiatric clinical trials, the phenomenon of conflicts of interest due to economic gains is relatively common, which is often associated with trial results that are highly favorable to the intervention measures (98). We suspect that researchers, owing to conflicts of interest, may be more inclined to publish positive results, thereby leading to publication bias. To minimize the impact of publication bias on the study results as much as possible, we made every effort to retrieve literature in various ways to reduce publication bias. For example, in addition to searching common academic databases (such as PubMed and Web of Science), we also searched clinical trial registration platforms (such as ClinicalTrials.gov), gray literature databases, and conference proceedings, and contacted study authors to obtain unpublished data. Although these measures greatly expanded the sources of literature and successfully included more studies that were originally unpublished or difficult to obtain, publication bias could not be completely eliminated. When contacting some authors, they refused to provide data for various reasons, which, to some extent, limited our control over publication bias and also suggests that more effective methods need to be further explored in future studies to deal with such challenges.

In the bias risk assessment of this study, we found significant issues that the majority of the included studies failed to address: adequately describing the randomization process and implementing blinding. This finding has important implications for the interpretation and application of our research results. Randomization is a key step in reducing selection bias, ensuring that the probability of subjects being assigned to different intervention groups is equal, thereby enhancing the internal validity of the study results (99). However, among the studies included in this review, 52 studies did not provide a detailed description of the randomization method. This omission makes it difficult for us to assess the scientific and rigorous nature of the randomization process in these studies. For example, some studies simply mentioned “random assignment” without specifying the exact randomization technique (such as using random number tables or computer random number generators). This vague description not only affects our judgment of the study quality but also increases the risk of the results being influenced by selection bias.

Blinding is an important means of reducing subjective bias (100), which includes the blinding of subjects, researchers, and outcome assessors. In the studies included in this review, 47 studies did not implement any form of blinding. We analyzed the reasons for this from three aspects. On the one hand, it is the particularity of the schizophrenia patient population. The symptom characteristics and cognitive impairments of patients with schizophrenia may interfere with the implementation of blinding (101). For example, patients’ hallucinations, delusions, and other symptoms may affect their objective perception and judgment of the intervention process; cognitive impairments may lead to difficulties for patients in understanding research requirements and following blinding instructions, thereby reducing the success rate of blinding. On the other hand, it is the role and influence of the therapist (102). Psychotherapy emphasizes the establishment of a good therapeutic relationship between the therapist and the patient. The process of establishing this relationship may itself reveal the nature of the intervention. During the treatment process, therapists need to communicate and interact deeply with patients to understand their inner world (103). This close relationship may make patients more likely to perceive the therapist’s intentions and the purpose of the intervention, thereby breaking the blinding. The third aspect is the difficulty in practical operation. In actual research operations, strictly implementing blinding requires a large number of resources and effort, including designing complex intervention plans, training therapists and research teams, and monitoring the research process (104). For some research teams with limited resources, this may be an insurmountable obstacle.

In this study, the quality of evidence for various outcome indicators showed significant differences, with the quality of evidence for mental health outcomes being rated as low. Although the study employed an RCT design, it faced serious risks of bias and inconsistency. The serious risk of bias is likely due to the ineffective implementation of randomization and blinding during the study execution (105). This leads to an imbalance between the experimental and control groups, thereby seriously interfering with the accuracy and reliability of the study results. The severe inconsistency indicates significant differences in the results among different studies, which may be rooted in the diverse characteristics of schizophrenia in the patients included in each study, as well as the distinct details of the interventions.

The quality of evidence for the wellbeing outcome is at an intermediate level. The assessment of this outcome indicator is affected by the flaws in the measurement tools or systematic biases in sample selection, making it difficult for the study results to accurately reflect the real situation. However, thanks to the absence of serious problems in inconsistency, indirectness, and imprecision, its quality of evidence is relatively higher compared to some other indicators.

The quality of evidence for social functioning outcomes is rated as very low. Even with the RCT design, the risks of bias, inconsistency, and other considerations are all in a serious state. The serious risk of bias may be attributed to significant flaws in the research methods, such as the lack of uniformity or accuracy in the methods used to assess social functioning.

The quality of evidence for social adaptability, positive symptoms, negative symptoms, PANSS-Total score, cognitive function, and self-esteem outcomes is also low. These indicators have problems of bias and imprecision. The serious risk of bias may be due to defects in the study design or improper data collection methods; the serious imprecision is probably due to insufficient sample size, leading to biased estimates of effect sizes.

At the same time, considering the individual differences among patients with schizophrenia (106, 107), future research should focus on how to tailor personalized positive psychological intervention plans according to the individual differences of patients in order to better meet the specific needs of different patients (97, 108, 109). Future research should conduct a comprehensive assessment of patients’ needs, obtaining information on patients’ needs from multiple dimensions. Based on the assessment results, a personalized treatment plan should be formulated for each patient. The treatment plan should include specific intervention goals, methods, frequency, and duration (110). For example, patients in the acute phase may require more frequent treatments and more intensive interventions; for patients in the stable phase, the treatment frequency can be gradually reduced, focusing on maintenance treatment and prevention of relapse (111). During the intervention process, regularly assess the patient’s progress and changes in needs, and flexibly adjust the intervention plan.

Recording patient progress at multiple time intervals is an important means of assessing the effectiveness of intervention measures and adjusting treatment plans (112). Electronic health record systems can be used to record and manage patient assessment data. EHR systems provide convenient functions for data entry, storage, and query, ensuring the security and integrity of the data (113). A dedicated research database can also be established to record patient assessment data and follow-up information. The database can perform data cleaning, statistical analysis, and visualization, facilitating researchers in data analysis and reporting (114).

Since this study only targets the Chinese population, it limits the generalizability of the research conclusions to a broader non-Chinese context. In the future, multicenter cross-cultural studies should be conducted, including patients from different regions and cultural backgrounds, to compare the effects and implementation experiences of personalized intervention measures across different cultural settings. Cross-cultural studies can provide references for schizophrenia treatment on a global scale, promote exchanges and cooperation between different cultures, and thereby enhance the universality and representativeness of the research findings (115).

In resource-limited environments, incorporating positive psychology interventions into clinical practice requires innovation and flexibility. Collaborate with community health service centers, non-governmental organizations (NGOs), volunteer groups, and other organizations to utilize existing community resources for intervention activities (116). For example, community health service centers can provide venues and basic facilities, and volunteers can assist in organizing and implementing intervention activities. Develop self-help intervention tools, such as self-help manuals and workbooks, which patients can use at home. These tools can provide structured guidance and exercises to help patients carry out self-interventions without the guidance of professionals. Utilize the internet and mobile technology to provide online intervention courses and resources. By developing mobile applications or online platforms, patients can access positive psychology intervention content at home. Train non-professionals, including community health workers, social workers, and volunteers, to enable them to assist in implementing some intervention measures. The training content can include basic mental health knowledge, intervention skills, and patient communication skills, among others. Train patient family members to support the patient’s intervention process. Family members can provide emotional support, supervise patients to complete intervention tasks, and participate in homework.

Ensure the sustainability of long-term treatment by adopting a long-term follow-up mechanism. Establish a long-term follow-up mechanism to regularly assess the patient’s treatment effectiveness and changes in needs and adjust the treatment plan in time. Follow-ups can be conducted in various forms, such as telephone, text messages, and online questionnaires, to improve the efficiency and coverage of follow-ups (117). Conduct cost-effectiveness analyses to evaluate the economic benefits of intervention measures. By optimizing resource allocation, ensure the sustainability of intervention measures in resource-limited environments (118). For example, by reducing unnecessary intervention links, the intervention cost can be reduced, and the efficiency of resource utilization can be improved.

### Challenges and limitations

In this meta-analysis, we have identified certain limitations in the study design and implementation that may have impacted our conclusions. Firstly, the large time span included in the study, coupled with regional differences, may have led to modifications of the scale. As a result, when using the PANSS for assessment, the heterogeneity of the measurement results is relatively high. In addition, when conducting subgroup analyses on wellbeing, mental health, positive symptoms, negative symptoms, and social functioning, we found no significant differences between medium-term and long-term treatments. This may be due to the different number of studies included in each group and the relatively small sample sizes. We also noted that most of the studies included did not provide detailed descriptions of their randomization methods, and blinding techniques were not employed, which may have affected the objectivity and accuracy of the results. Furthermore, the necessity for personalized treatment to adapt to the patients’ evolving needs and therapeutic responses may have led to inconsistencies in treatment outcomes, thereby affecting the stability of the study. The implementation of long-term treatment also faces challenges such as resource constraints, low patient engagement, and questions of treatment sustainability.

Regarding the issue of publication bias. For the indicators of social functioning as well as positive and negative symptoms, the detected publication bias poses a challenge to the robustness and generalizability of our research results. The asymmetry presented by the funnel plot and the results of Egger’s test strongly suggest that the existing literature may not cover all the studies conducted in this field. It is highly likely that studies with non-significant or negative results have not been published, while those with desirable outcomes are more likely to enter the public eye. This selective dissemination of research findings can lead to a biased overall situation, thereby causing an overestimation or underestimation of the true effect sizes related to improvements in social functioning and changes in symptoms.

## Conclusion

Positive psychology has significant effects on enhancing the wellbeing of Chinese patients with schizophrenia. It not only improves the positive and negative symptoms of the disorder but also strengthens social adaptability and cognitive functions. Moreover, positive psychology provides clear benefits in alleviating depressive symptoms among individuals with schizophrenia. Notably, the long-term adherence to positive psychological interventions yields much better treatment outcomes than short-term interventions. Therefore, we recommend the widespread application of positive psychology in clinical treatment.

## Data Availability

The datasets presented in this study can be found in online repositories. The names of the repository/repositories and accession number(s) can be found in the article/**Supplementary Material**.

## References

[1] Kharawala S Hastedt C Podhorna J Shukla H Kappelhoff B Harvey PD . The relationship between cognition and functioning in schizophrenia: A semi-systematic review, Schizophrenia research. Cognition. (2022) 27:100217. doi: 10.1016/j.scog.2021.100217 PMC8488595 34631435

[2] Correll CU Martin A Patel C Benson C Goulding R Kern-Sliwa J . Systematic literature review of schizophrenia clinical practice guidelines on acute and maintenance management with antipsychotics. Schizophr (Heidelberg Germany). (2022) 8:5. doi: 10.1038/s41537-021-00192-x PMC8873492 35210430

[3] Rivelli A Fitzpatrick V Nelson M Laubmeier K Zeni C Mylavarapu S . Real-world predictors of relapse in patients with schizophrenia and schizoaffective disorder in a large health system. Schizophr (Heidelberg Germany). (2024) 10:28. doi: 10.1038/s41537-024-00496-8 PMC10904733 38424086

[4] Nowak I Sabariego C Świtaj P Anczewska M . Disability and recovery in schizophrenia: a systematic review of cognitive behavioral therapy interventions. BMC Psychiatry. (2016) 16:228. doi: 10.1186/s12888-016-0912-8 27400680 PMC4940955

[5] Molstrom IM Nordgaard J Urfer-Parnas A Handest R Berge J Henriksen MG . The prognosis of schizophrenia: A systematic review and meta-analysis with meta-regression of 20-year follow-up studies. Schizophr Res. (2022) 250:152–63. doi: 10.1016/j.schres.2022.11.010 36417817

[6] Kassebaum NJ Arora M Barber RM Bhutta ZA Brown J Carter A . Global, regional, and national disability-adjusted life-years (DALYs) for 315 diseases and injuries and healthy life expectancy (HALE), 1990–2015: a systematic analysis for the Global Burden of Disease Study 2015. Lancet. (2016) 388:1603–58. doi: 10.1016/S0140-6736(16)31460-X PMC5388857 27733283

[7] Solmi M Radua J Olivola M Croce E Soardo L Salazar de Pablo G . Age at onset of mental disorders worldwide: large-scale meta-analysis of 192 epidemiological studies. Mol Psychiatry. (2022) 27:281–95. doi: 10.1038/s41380-021-01161-7 PMC8960395 34079068

[8] Charlson FJ Ferrari AJ Santomauro DF Diminic S Stockings E Scott JG . Global epidemiology and burden of schizophrenia: findings from the global burden of disease study 2016. Schizophrenia Bull. (2018) 44:1195–203. doi: 10.1093/schbul/sby058 PMC6192504 29762765

[9] Chong HY Teoh SL Wu DB Kotirum S Chiou CF Chaiyakunapruk N . Global economic burden of schizophrenia: a systematic review. Neuropsychiatr Dis Treat. (2016) 12:357–73. doi: 10.2147/NDT.S96649 PMC4762470 26937191

[10] Qiu D Li Y Wu Q An Y Tang Z Xiao S . Patient’s disability and caregiver burden among Chinese family caregivers of individual living with schizophrenia: mediation effects of potentially harmful behavior, affiliate stigma, and social support. Schizophr (Heidelberg Germany). (2023) 9:83. doi: 10.1038/s41537-023-00418-0 PMC10692118 38040711

[11] Huang Y Wang Y Wang H Liu Z Yu X Yan J . Prevalence of mental disorders in China: a cross-sectional epidemiological study. Lancet Psychiatry. (2019) 6:211–24. doi: 10.1016/S2215-0366(18)30511-X 30792114

[12] G. M. D. C. J. T. L. Psychiatry . Global, regional, and national burden of 12 mental disorders in 204 countries and territories, 1990–2019: a systematic analysis for the Global Burden of Disease Study 2019. Lancet Psych. (2022) 9:137–50. doi: 10.1016/S2215-0366(21)00395-3 PMC8776563 35026139

[13] Luvsannyam E Jain MS Pormento MKL Siddiqui H Balagtas ARA Emuze BO . Neurobiology of schizophrenia: A comprehensive review. Cureus. (2022) 14:e23959. doi: 10.7759/cureus.23959 35541299 PMC9080788

[14] Solmi M Croatto G Piva G Rosson S Fusar-Poli P Rubio JM . Efficacy and acceptability of psychosocial interventions in schizophrenia: systematic overview and quality appraisal of the meta-analytic evidence. Mol Psychiatry. (2023) 28:354–68. doi: 10.1038/s41380-022-01727-z 35999275

[15] Leijala J Kampman O Suvisaari J Eskelinen S . Daily functioning and symptom factors contributing to attitudes toward antipsychotic treatment and treatment adherence in outpatients with schizophrenia spectrum disorders. BMC Psychiatry. (2021) 21:37. doi: 10.1186/s12888-021-03037-0 33441112 PMC7805157

[16] Chow RTS Whiting D Favril L Ostinelli E Cipriani A Fazel S . An umbrella review of adverse effects associated with antipsychotic medications: the need for complementary study designs. Neurosci Biobehav Rev. (2023) 155:105454. doi: 10.1016/j.neubiorev.2023.105454 37925094 PMC10914636

[17] Read J Williams J . Positive and negative effects of antipsychotic medication: an international online survey of 832 recipients. Curr Drug Saf. (2019) 14:173–81. doi: 10.2174/1574886314666190301152734 PMC6864560 30827259

[18] Stevović LI Repišti S Radojičić T Sartorius N Tomori S Kulenović AD . Non-pharmacological interventions for schizophrenia-analysis of treatment guidelines and implementation in 12 Southeast European countries. Schizophr (Heidelberg Germany). (2022) 8:10. doi: 10.1038/s41537-022-00226-y PMC8888596 35232972

[19] Chen LF Liu J Zhang J Lu XQ . Non-pharmacological interventions for caregivers of patients with schizophrenia: A meta-analysis. Psychiatry Res. (2016) 235:123–7. doi: 10.1016/j.psychres.2015.11.037 26639649

[20] Seligman ME Csikszentmihalyi M . Positive psychology. An introduction. Am Psychol. (2000) 55:5–14. doi: 10.1037/0003-066X.55.1.5 11392865

[21] Chakhssi F Kraiss JT Sommers-Spijkerman M Bohlmeijer ET . The effect of positive psychology interventions on well-being and distress in clinical samples with psychiatric or somatic disorders: a systematic review and meta-analysis. BMC Psychiatry. (2018) 18:211. doi: 10.1186/s12888-018-1739-2 29945603 PMC6020379

[22] Fan CH Hsu SC Hsiao FH Chang CM Liu CY Lai YM . The association of social support and symptomatic remission among community-dwelling schizophrenia patients: A cross-sectional study. Int J Environ Res Public Health. (2021) 18. doi: 10.3390/ijerph18083977 PMC8070210 33918873

[23] Schrank B Brownell T Jakaite Z Larkin C Pesola F Riches S . Evaluation of a positive psychotherapy group intervention for people with psychosis: pilot randomised controlled trial. Epidemiol Psychiatr Sci. (2016) 25:235–46. doi: 10.1017/S2045796015000141 PMC6998731 25698298

[24] Kang SJ Ko SH Kim JY Kim SR . Effects of a mental fitness positive psychology intervention program on inpatients with schizophrenia in South Korea: A feasibility study. Perspect Psychiatr Care. (2020) 56:6–13. doi: 10.1111/ppc.12332 30430580

[25] Favrod J Nguyen A Fankhauser C Ismailaj A Hasler JD Ringuet A . Positive Emotions Program for Schizophrenia (PEPS): a pilot intervention to reduce anhedonia and apathy. BMC Psychiatry. (2015) 15:231. doi: 10.1186/s12888-015-0610-y 26419356 PMC4588492

[26] Meyer PS Johnson DP Parks A Iwanski C Penn DL . Positive living: A pilot study of group positive psychotherapy for people with schizophrenia. J Positive Psychol. (2012) 7:239–48. doi: 10.1080/17439760.2012.677467

[27] Kim J Na H . Effects of a positive psychotherapy program on positive affect, interpersonal relations, resilience, and mental health recovery in community-dwelling people with schizophrenia. JKAN. (2017) 47:638–50. doi: 10.4040/jkan.2017.47.5.638 29151561

[28] Pina I Braga CM de Oliveira TFR de Santana CN Marques RC MaChado L . Positive psychology interventions to improve well-being and symptoms in people on the schizophrenia spectrum: a systematic review and meta-analysis. Rev Bras psiquiatria (Sao Paulo Brazil: 1999). (2021) 43:430–7. doi: 10.1590/1516-4446-2020-1164 PMC8352741 33331497

[29] O’Connor D Green S Higgins JP . Defining the review question and developing criteria for including studies. Cochrane Handb Syst Rev Interv. (2008), 81–94.

[30] Cumpston M Li T Page MJ Chandler J Welch VA Higgins JP . Updated guidance for trusted systematic reviews: a new edition of the Cochrane Handbook for Systematic Reviews of Interventions. (2019) 2019:. doi: 10.1002/14651858.ED000142 PMC10284251 31643080

[31] Higgins JP Altman DG Gøtzsche PC Jüni P Moher D Oxman AD . The Cochrane Collaboration’s tool for assessing risk of bias in randomised trials. BMJ (Clinical Res ed.). (2011) 343:d5928. doi: 10.1136/bmj.d5928 PMC3196245 22008217

[32] Guyatt GH Oxman AD Kunz R Vist GE Falck-Ytter Y Schünemann HJJB . What is “quality of evidence” and why is it important to clinicians? BMJ. (2008) 336:995–8. doi: 10.1136/bmj.39490.551019.BE PMC2364804 18456631

[33] Guyatt GH Oxman AD Kunz R Brozek J Alonso-Coello P Rind D . GRADE guidelines 6. Rating the quality of evidence—imprecision. Journal of Clinical Epidemiology (2011) 64:1283–93. doi: 10.1016/j.jclinepi.2011.01.012 21839614

[34] Moher D Liberati A Tetzlaff J Altman DG . Preferred reporting items for systematic reviews and meta-analyses: the PRISMA statement. PloS Med. (2009) 6:e1000097. doi: 10.1371/journal.pmed.1000097 19621072 PMC2707599

[35] Egger M Davey Smith G Schneider M Minder C . Bias in meta-analysis detected by a simple, graphical test. BMJ (Clinical Res ed.). (1997) 315:629–34. doi: 10.1136/bmj.315.7109.629 PMC2127453 9310563

[36] Aixia J . Nursing intervention based on ‘Positive Psychology Theory’ in patients with schizophrenia. J J Qilu Nurs. (2015) 21:20–21,22. doi: 10.3969/j.issn.1006-7256.2015.15.007

[37] Lin F . Nursing effect analysis of positive psychology theory in clinical rehabilitation of schizophrenia. J Gen Pract Nurs. (2015) 13:3366–8. doi: 10.3969/j.issn.1674-4748.2015.33.023

[38] Ni J Zhu C Lu Q . A study on the effect of nursing intervention based on positive psychology theory on the recovery process of schizophrenia patients. J J North Sichuan Med Coll. (2019) 34:148–51. doi: 10.3969/j.issn.1005-3697.2019.01.041

[39] He J . Exploring the nursing effect of positive psychology theory in the clinical rehabilitation of patients with schizophrenia. J Contemp Nurses (Lower Decade) J Today Nurse. (2020) 27:125–6. doi: 10.19793/j.cnki.1006-6411.2020.18.052

[40] Shu L Cao Y Shi Y . Effectiveness of positive psychology theory in rehabilitation care for schizophrenia. J Med Inf. (2015) 28:193–4. doi: 10.3969/j.issn.1006-1959.2015.45.265

[41] Chen L Zhuo R Zheng G Nina H Zheng C . A study on the application of positive psychology theory in clinical rehabilitation of schizophrenia. J Qiqihar Univ Med. (2015) 36:5268–9.

[42] Cai M . An analysis of the application of positive psychology theory in rehabilitation care for patients with schizophrenia. J Chin Community Doctors. (2022) 38:105–7. doi: 10.3969/j.issn.1007-614x.2022.05.035

[43] Cao N Xu X . Clinical effectiveness of applying positive psychology theory in schizophrenia rehabilitation nursing care. J Clin Res Pract. (2017) 2:169–70. doi: 10.19347/j.cnki.2096-1413.201722084

[44] Cao S . The role of positive psychology theory in rehabilitation care for patients with schizophrenia. Heilongjiang J Traditional Chin Med. (2021) 50:185–6.

[45] Yuan S Zhu C Zhang K Shen C . A study of the effect of nursing intervention guided by positive psychology theory on the recovery and social functioning of patients with schizophrenia. J Hosp Manage Forum. (2020) 37:40–43,47. doi: 10.3969/j.issn.1671-9069.2020.08.012

[46] Hu X Wu H Wu Q . Analysis of the effect of applying positive psychology theory rehabilitation nursing in patients with schizophrenia. Front Med J Yiyao Qianyan. (2019) 9:154.

[47] Lai SY . The effect of positive psychology theory in the rehabilitation nursing of schizophrenia. Chin Sci Technol J Database (Full Text Edition) Med Health. (2022) 43:219–22.

[48] Gu XH Zhao HY Kong FZ . Exploring the effects of positive psychology theory in the rehabilitation of patients with schizophrenia. J Chin Community Doctors. (2021) 37:140–1. doi: 10.3969/j.issn.1007-614x.2021.30.069

[49] Liu X-Y . Effectiveness analysis of positive psychology theory-based nursing care for patients with schizophrenia. J Contemp Nurses (in Chinese). (2016) 11:78–80.

[50] Uyahan . Positive psychology theory in clinical rehabilitation of schizophrenia. J World Latest Med Inf Abstracts (Continuous Electronic Journal). (2016) 16:109. doi: 10.3969/j.issn.1671-3141.2016.82.088

[51] Fang Y Li F Liao J . Observing the effect of applying positive psychology theory to implement rehabilitation nursing care for patients with schizophrenia. Med J Thepresent Clin. (2022) 35:74–5. doi: 10.3969/j.issn.2095-9559.2022.02.44

[52] Shen Y . Effective application of positive psychology theory in rehabilitation nursing care for patients with schizophrenia. J Chin Foreign Med Res. (2018) 16:97–8. doi: 10.14033/j.cnki.cfmr.2018.26.044

[53] Xu Y . Nursing intervention guided by positive psychology theory in schizophrenia rehabilitation. J Med Theory Pract. (2018) 31:2674–6. doi: 10.19381/j.issn.1001-7585.2018.17.080

[54] Feng Y . Effectiveness of positive psychology in rehabilitation care for patients with schizophrenia. J Chin Remedies Clinics. (2019) 19:3816–8. doi: 10.11655/zgywyle2019.21.087

[55] Zhang YW . Effectiveness of positive psychology assessment applied to rehabilitation care of schizophrenia. J Chin Community Physician. (2023) 39:130–2. doi: 10.3969/j.issn.1007-614x.2023.04.044

[56] Li Z Han P Qiu A . Effectiveness of positive psychology theory in rehabilitation nursing of schizophrenia. Chin J Nurs. (2013) 48:1098–101. doi: 10.3761/j.issn.0254-1769.2013.12.015

[57] Xie J Tan F Zhao L Liu Z Long M . The value of positive psychology theory in rehabilitation nursing care for schizophrenia. J Reflexol Rehabil Med. (2021) 2:125–127,134.

[58] Liu J . Effectiveness of positive psychology theory in schizophrenia rehabilitation nursing care. J Guide China Med. (2018) 16:247–8. doi: 10.15912/j.cnki.gocm.2018.07.208

[59] Ma M Xi’an S . (2023). China.

[60] Tingting T . Exploring the effect of positive psychology theory in rehabilitation care for schizophrenia. J Capital Food Med J Capital Med. (2019) 26:158.

[61] Zhao W . Analysing the effect of positive psychology theory in the rehabilitation care of schizophrenia. Electronic J Pract Clin Nurs. (2016) 1:128–128,131.

[62] Liu X . Analysis of the application effect of positive psychology theory in the rehabilitation nursing of schizophrenia. China Sci Technol J Database Med. (2022) 5:21–124.

[63] Wang X . Analysis of the effect of applying positive psychology theory in clinical rehabilitation of schizophrenia. J Med Inf. (2017) 30:73–4.

[64] Liang X-L Li Z-C Lu E-Y . Effects of rehabilitation nursing based on positive psychology theory on the sense of well-being and enterprising and health status of patients with schizophrenia. J Chin Clin Nurs. (2019) 11:339–42. doi: 10.3969/j.issn.1674-3768.2019.04.019

[65] Yu Y . Exploring the nursing effect of positive psychology theory in clinical rehabilitation of patients with schizophrenia. J Primary Care Med Forum J Med Forum. (2020) 24:836–7. doi: 10.19435/j.1672-1721.2020.06.062

[66] Zhu Y Liao X Zhang X Liu S Huang C Du S . Effectiveness of the application of positive psychology theory in rehabilitation nursing care for schizophrenia. J China Modern Med. (2021) 28:244–6.

[67] Liu Z . Positive psychology theory for schizophrenia rehabilitation nursing effect evaluation. J Chin Pharm Industry. (2021) 30:192–3. doi: 10.3969/j.issn.1006-4931.2021.Z1.149

[68] Li B . Evaluation of the value of positive psychology theory applied to schizophrenia rehabilitation nursing care. J Chin Foreign Women’s Health Res J Women’s Health Res. (2019) 7:19–20.

[69] Wu X Zheng L . The effect of nursing intervention based on positive psychology theory on the recovery process of patients with schizophrenia. J Nurs Integrated Traditional Chin Western Med. (2021) 7:148–50.

[70] Fu M . Analysing the application of positive psychology theory in rehabilitation nursing care for schizophrenia and its impact on ADL scores. Chin Sci Technol J Database (Citation Edition) Med Health. (2022) 9:173–5.

[71] Wang JP . Application of positive psychology theory in schizophrenia rehabilitation nursing care. Chin Sci Technol J Database (Digest Edition) Med Health. (2022) 11:145–7.

[72] Li A Hong A Wen D . Effects of a nursing model based on positive psychology theory on female patients with schizophrenia in terms of stigma and psychological status. J Heilongjiang Med. (2023) 47:368–70. doi: 10.3969/j.issn.1004-5775.2023.03.034

[73] Xu L . The effect of nursing intervention of positive psychology theory on psychological status and sense of shame of schizophrenic patients. J Weekly Digest - Elderly Weekly. (2023) 17:221–3.

[74] Xing H . The application effect of positive psychology theory in the rehabilitation nursing of schizophrenia. Chin Sci Technol J Database (Digest Edition) Med Health. (2023) 9:130–2.

[75] Fang Y Xu H Zhou W . Intervention effects of psychiatric rehabilitation nursing based on positive psychology theory in patients with schizophrenia. J Psychol Monthly. (2024) 19:64–6. doi: 10.19738/j.cnki.psy.2024.05.020

[76] Wu C . Effectiveness of positive psychology theory in rehabilitation care for schizophrenia. J China Continuing Med Educ. (2015) 7:237–8. doi: 10.3969/j.issn.1674-9308.2015.23.171

[77] Fan Y Xian Y Li N . The value of nursing intervention guided by positive psychology theory for patients with schizophrenia. J Hainan Med J. (2016) 27:342–4.

[78] Bai G . Positive psychology in rehabilitation nursing care for patients with schizophrenia. Chin J Urban Rural Enterprise Hygiene. (2019) 34:218–9. doi: 10.16286/j.1003-5052.2019.07.089

[79] Lu J Rong C Xu X Lai Y . Effects of psychological care under the guidance of positive psychology on patients with schizophrenia. J Qilu Nurs. (2019) 25:109–11. doi: 10.3969/j.issn.1006-7256.2019.09040

[80] Zhou J . The effects of nursing interventions guided by positive psychology on social functioning and self-esteem levels of patients with schizophrenia. J Primary Care Med Forum J Med Forum. (2020) 24:3484–5. doi: 10.19435/j.1672-1721.2020.24.046

[81] Tang N Yuan P Liao Y Zeng WX Guo XY . The value of positive psychology in the care of elderly patients with schizophrenia. J China Modern Med. (2020) 27:242–4.

[82] Gu J Wang X . Effect of positive psychology theory nursing in the rehabilitation of patients with schizophrenia. J Chin People’s Health Med Med J Chin People’s Health. (2021) 33:84–6. doi: 10.3969/j.issn.1672-0369.2021.04.033

[83] Wu Y Gong C . The effects of positive psychology-based rehabilitation nursing on coping styles and social functioning in patients with schizophrenia. J Chin Clin Nurs. (2024) 16:100–3. doi: 10.3969/j.issn.1674-3768.2024.02.007

[84] Li Z Du D Yang J Liu S . An analysis of the effects of nursing interventions applying positive psychology theory on the psychological status and sense of shame of schizophrenia patients. Front Med J Yiyao Qianyan. (2021) 11:132–133,136.

[85] Chen J Nursing Practice J . Application of positive psychology theory in rehabilitation nursing care for patients with schizophrenia. Res. (2016) 13:130–1. doi: 10.3969/j.issn.1672-9676.2016.23.057

[86] Xu J . Study on the effect of the application of positive psychology theory in rehabilitative care for patients with schizophrenia. J Contemp Med Forum. (2018) 16:268–9.

[87] Wang Y . Analysis of the impact of positive psychology theory on cognitive and social competence of patients with schizophrenia. J Contemp Med. (2019) 25:8–10.

[88] Yu L-H . The role of positive psychology theory in reducing relapse rate of schizophrenic patients in rehabilitation nursing care. Chin Sci Technol J Database (Full Text Edition) Med Health. (2021) 2:179–81.

[89] Shi CL . Evaluation of the effect of positive psychology theory on rehabilitation nursing care for patients with schizophrenia. Chin Sci Technol J Database (Citation Edition) Med Health. (2022) 5:270–3.

[90] Chandler J Cumpston M Li T Page MJ Welch VJHW . Cochrane handbook for systematic reviews of interventions. Cochrane Datab Syst Rev. (2019). doi: 10.1002/9781119536604 PMC10284251 31643080

[91] Weittenhiller LP Mikhail ME Mote J Campellone TR Kring AM . What gets in the way of social engagement in schizophrenia? World J Psychiatry. (2021) 11:13–26. doi: 10.5498/wjp.v11.i1.13 33511043 PMC7805250

[92] Wang Q Zhu X Jiang X Li M Chang R Chen B . Relationship between stressful life events, coping styles, and schizophrenia relapse. Int J Ment Health Nurs. (2021) 30:1149–59. doi: 10.1111/inm.12865 PMC8518398 33960095

[93] De Berardis D Vellante F Olivieri L Rapini G De Lauretis I Orsolini L . The effect of paliperidone palmitate long-acting injectable (PP-LAI) on “non-core” symptoms of schizophrenia: a retrospective, collaborative, multicenter study in the “real world” everyday clinical practice. Rivista di psichiatria. (2021) 56:143–8. doi: 10.1708/3635.36155 34196631

[94] Sampogna G Di Vincenzo M Giuliani L Menculini G Mancuso E Arsenio E . A systematic review on the effectiveness of antipsychotic drugs on the quality of life of patients with schizophrenia. Brain Sci. (2023) 13:1577. doi: 10.3390/brainsci13111577 38002537 PMC10669728

[95] Bolier L Haverman M Westerhof GJ Riper H Smit F Bohlmeijer E . Positive psychology interventions: a meta-analysis of randomized controlled studies. BMC Public Health. (2013) 13:119. doi: 10.1186/1471-2458-13-119 23390882 PMC3599475

[96] Kart A Özdel K Türkçapar MH . Cognitive behavioral therapy in treatment of schizophrenia. Noro psikiyatri arsivi. (2021) 58:S61–s65. doi: 10.5152/npa.2021.66300 34658637 PMC8498814

[97] McDonagh MS Dana T Kopelovich SL Monroe-DeVita M Blazina I Bougatsos C . Psychosocial interventions for adults with schizophrenia: an overview and update of systematic reviews. Psychiatr Serv (Washington D.C.). (2022) 73:299–312. doi: 10.1176/appi.ps.202100334 34384230

[98] Lundh A Lexchin J Mintzes B Schroll JB Bero L . Industry sponsorship and research outcome. Cochrane Database Syst Rev. (2017) 2:Mr000033. doi: 10.1002/14651858.MR000033.pub3 28207928 PMC8132492

[99] Rosenkranz GK . The impact of randomization on the analysis of clinical trials. Stat Med. (2011) 30:3475–87. doi: 10.1002/sim.v30.30 21953285

[100] Day SJ Altman DG . Blinding in clinical trials and other studies. BMJ. (2000) 321:504. doi: 10.1136/bmj.321.7259.504 10948038 PMC1118396

[101] Kang D Zhang Y Wu G Song C Peng X Long Y . The effect of accelerated continuous theta burst stimulation on weight loss in overweight individuals with schizophrenia: A double-blind, randomized, sham-controlled clinical trial. Schizophr Bull. (2023) 50:589–99. doi: 10.1093/schbul/sbad144 PMC11059792 37921353

[102] Juul S Gluud C Simonsen S Frandsen FW Kirsch I Jakobsen JC . Blinding in randomised clinical trials of psychological interventions: a retrospective study of published trial reports. BMJ Evidence-Based Med. (2021) 26:109. doi: 10.1136/bmjebm-2020-111407 32998993

[103] Wright JH Davis D . The therapeutic relationship in cognitive-behavioral therapy: Patient perceptions and therapist responses. Cogn Behav Pract. (1994) 1:25–45. doi: 10.1016/S1077-7229(05)80085-9

[104] Monaghan TF Agudelo CW Rahman SN Wein AJ Lazar JM Everaert K . Blinding in clinical trials: seeing the big picture. Medicina (Kaunas Lithuania). (2021) 2021:57. doi: 10.3390/medicina57121373 PMC8308085 34202486

[105] Mansournia MA Higgins JP Sterne JA Hernán MA . Biases in randomized trials: A conversation between trialists and epidemiologists. Epidemiol (Cambridge Mass.). (2017) 28:54–9. doi: 10.1097/EDE.0000000000000576 PMC5130591 27748683

[106] Fan Y Tao Y Wang J Gao Y Wei W Zheng C . Irregularity of visual motion perception and negative symptoms in schizophrenia. Schizophr (Heidelberg Germany). (2024) 10:82. doi: 10.1038/s41537-024-00496-8 PMC11443095 39349502

[107] Rolls ET Lu W Wan L Yan H Wang C Yang F . Individual differences in schizophrenia. BJPsych Open. (2017) 3:265–73. doi: 10.1192/bjpo.bp.117.005058 PMC5676076 29163982

[108] Jeste DV Palmer BW Saks ER . Why we need positive psychiatry for schizophrenia and other psychotic disorders. Schizophr Bull. (2017) 43:227–9. doi: 10.1093/schbul/sbw184 PMC5605254 28399307

[109] Chien WT Leung SF Yeung FK Wong WK . Current approaches to treatments for schizophrenia spectrum disorders, part II: psychosocial interventions and patient-focused perspectives in psychiatric care. Neuropsychiatr Dis Treat. (2013) 9:1463–81. doi: 10.2147/NDT.S49263 PMC3792827 24109184

[110] Vita A Fagiolini A Maina G Mencacci C Spina E Galderisi S . Achieving long-term goals through early personalized management of schizophrenia: expert opinion on the role of a new fast-onset long-acting injectable antipsychotic. Ann Gen Psychiatry. (2023) 22:1. doi: 10.1186/s12991-022-00430-1 36650545 PMC9843844

[111] Besana F Civardi SC Mazzoni F Carnevale Miacca G Arienti V Rocchetti M . Predictors of readmission in young adults with first-episode psychosis: A multicentric retrospective study with a 12-month follow-up. Clinics Pract. (2024) 14:1234–44. doi: 10.3390/clinpract14040099 PMC11270315 39051293

[112] Jansen A-JS Peters GM Kooij L Doggen CJM van Harten WH . Device based monitoring in digital care and its impact on hospital service use. NPJ Digital Med. (2025) 8:16. doi: 10.1038/s41746-024-01427-8 PMC11711286 39779761

[113] Ozonze O Scott PJ Hopgood AA . Automating electronic health record data quality assessment. J Med Syst. (2023) 47:23. doi: 10.1007/s10916-022-01892-2 36781551 PMC9925537

[114] Aminizadeh S Heidari A Toumaj S Darbandi M Navimipour NJ Rezaei M . The applications of machine learning techniques in medical data processing based on distributed computing and the Internet of Things. Comput Methods Programs Biomed. (2023) 241:107745. doi: 10.1016/j.cmpb.2023.107745 37579550

[115] Myers NL . Update: schizophrenia across cultures. Curr Psychiatry Rep. (2011) 13:305–11. doi: 10.1007/s11920-011-0208-0 21643686

[116] Castillo EG Ijadi-Maghsoodi R Shadravan S Moore E Mensah MO III Docherty M . Community interventions to promote mental health and social equity. Focus: J Life Long Learn Psych. (2020) 18:60–70. doi: 10.1176/appi.focus.18102 PMC6996071 32015729

[117] Wold KF Kreis IV Åsbø G Flaaten CB Widing L Engen MJ . Long-term clinical recovery and treatment resistance in first-episode psychosis: a 10-year follow-up study. Schizophrenia. (2024) 10:69. doi: 10.1038/s41537-024-00489-7 39174576 PMC11341913

[118] Greenhawt M Oppenheimer J Codispoti CD . A practical guide to understanding cost-effectiveness analyses. J Allergy Clin Immunol: In Pract. (2021) 9:4200–7. doi: 10.1016/j.jaip.2021.10.006 34637929

