# Supplementary material for: The effects of positive psychology theory in the rehabilitation nursing of Chinese patients with schizophrenia: a systematic review and meta-analysis of randomized controlled trials
Source: Front Psychiatry. 2025 Feb 19;16:1515028. doi: 10.3389/fpsyt.2025.1515028 (PMC11880031; doi:10.3389/fpsyt.2025.1515028)
Supplement: Supplementary file 1 [file DataSheet1.zip › Data sheet 1/S1 Search strategy.docx.docx]

| **Database** | **Search Strategy** | **Results** |
| --- | --- | --- |
|  | **CNKI** |  |
|  | (Topic: Schizophrenia) AND (Topic: Positive Psychology) AND (Full text: Random (Precision)) | 59 |
|  | **Wan Fang** |  |
|  | Subject:(‘Schizophrenia’) and Subject:(‘Positive Psychology’) and All:(‘Random’) | 126 |
|  | **VIP** |  |
|  | ((Title or Keyword = Schizophrenia AND Title or Keyword = Positive Psychology) AND Any Field = Random) | 78 |
|  | **CBM** |  |
|  | ((‘Schizophrenia’ [weighted:not expanded] AND ‘Positive Psychology’ [weighted:not expanded] AND ‘Stochastic’ [all fields:Intelligence])) AND ( Human [feature word]) | 61 |
|  | **Pubmed** |  |
| #1 | "Schizophrenia"[Mesh] | 118353 |
| #2 | ((((((Schizophrenias[Title/Abstract]) OR (Dementia Praecox[Title/Abstract])) OR (Schizophrenic Disorders[Title/Abstract])) OR (Disorder, Schizophrenic[Title/Abstract])) OR (Disorders, Schizophrenic[Title/Abstract])) OR (Schizophrenic Disorder[Title/Abstract])) OR (schizophrenic syndrome[Title/Abstract]) | 2416 |
| #3 | ("Schizophrenia"[Mesh]) OR (((((((Schizophrenias[Title/Abstract]) OR (Dementia Praecox[Title/Abstract])) OR (Schizophrenic Disorders[Title/Abstract])) OR (Disorder, Schizophrenic[Title/Abstract])) OR (Disorders, Schizophrenic[Title/Abstract])) OR (Schizophrenic Disorder[Title/Abstract])) OR (schizophrenic syndrome[Title/Abstract])) | 118888 |
| #4 | "Psychology, Positive"[Mesh] | 189 |
| #5 | Positive Psychology | 174665 |
| #6 | ("Psychology, Positive"[Mesh]) OR (Positive Psychology) | 174665 |
| #7 | "Randomized Controlled Trial" [Publication Type] | 624,778 |
| #8 | ((((randomized controlled trial) OR (randomized)) OR (randomly)) OR (trial)) OR (groups) | 7,587,692 |
| #9 | ("Randomized Controlled Trial" [Publication Type]) OR (((((randomized controlled trial) OR (randomized)) OR (randomly)) OR (trial)) OR (groups)) | 7,587,692 |
| #10 | ((("Schizophrenia"[Mesh]) OR (((((((Schizophrenias[Title/Abstract]) OR (Dementia Praecox[Title/Abstract])) OR (Schizophrenic Disorders[Title/Abstract])) OR (Disorder, Schizophrenic[Title/Abstract])) OR (Disorders, Schizophrenic[Title/Abstract])) OR (Schizophrenic Disorder[Title/Abstract])) OR (schizophrenic syndrome[Title/Abstract]))) AND (("Psychology, Positive"[Mesh]) OR (Positive Psychology))) AND (("Randomized Controlled Trial" [Publication Type]) OR (((((randomized controlled trial) OR (randomized)) OR (randomly)) OR (trial)) OR (groups))) | 4013 |
|  | **Web of Science core collection** |  |
| #1 | TS=(Schizophrenia or Schizophrenias or Dementia Praecox or Schizophrenic Disorders or Disorder, Schizophrenic or Disorders, Schizophrenic or Schizophrenic Disorder or schizophrenic syndrome) | 185474 |
| #2 | TS=(Psychology, Positive or Positive Psychology) | 20114 |
| #3 | TS=(randomized controlled trial OR randomized OR randomly OR trial) | 2535604 |
| #4 | #1 AND #2 AND #3 | 31 |
|  | **Embase** |  |
| #1 | 'schizophrenia'/exp | 230260 |
| #2 | 'schizophrenias':ab,ti OR 'dementia  praecox':ab,ti OR 'schizophrenic disorders':ab,ti  OR 'disorder, schizophrenic':ab,ti OR 'disorders,  schizophrenic':ab,ti OR 'schizophrenic  disorder':ab,ti OR 'schizophrenic syndrome':ab,ti | 3077 |
| #3 | #1 OR #2 | 230564 |
| #4 | 'positive psychology'/exp | 798 |
| #5 | 'psychology, positive':ab,ti | 43 |
| #6 | #4 OR #5 | 822 |
| #7 | #3 AND #6 | 5 |
|  | **CINAHL Complete** |  |
| S1 | (MH "Schizophrenia") | 30102 |
| S2 | Schizophrenias OR Dementia Praecox OR Schizophrenic Disorders OR Disorder, Schizophrenic OR Disorders, Schizophrenic OR Schizophrenic Disorder | 39636 |
| S3 | S1 OR S2 | 39636 |
| S4 | (MH "Positive Psychology") | 2696 |
| S5 | Psychology, Positive | 10234 |
| S6 | S4 OR S5 | 10234 |
| S7 | (MH "Randomized Controlled Trials") | 147971 |
| S8 | randomized controlled trial OR randomized OR randomly OR trial | 694921 |
| S9 | S7 OR S8 | 694,921 |
| S10 | S3 AND S6 AND S9 | 202 |
|  | **APA PsycInfo (EBSCO)** |  |
| S1 | DE "Schizophrenia" | 109853 |
| S2 | Schizophrenias OR Dementia Praecox OR Schizophrenic Disorders OR Disorder, Schizophrenic OR Disorders, Schizophrenic OR Schizophrenic Disorder | 170663 |
| S3 | S1 OR S2 | 170663 |
| S4 | DE "Positive Psychology" | 6547 |
| S5 | Psychology, Positive | 243992 |
| S6 | S4 OR S5 | 243992 |
| S7 | DE "Randomized Controlled Trials" | 1110 |
| S8 | randomized controlled trial OR randomized OR randomly OR trial | 334875 |
| S9 | S7 OR S8 | 334875 |
| S10 | S3 AND S6 AND S9 | 2233 |
|  | **MEDLINE (EBSCO)** |  |
| S1 | (MH "Schizophrenia") | 114,272 |
| S2 | Schizophrenias OR Dementia Praecox OR Schizophrenic Disorders OR Disorder, Schizophrenic OR Disorders, Schizophrenic OR Schizophrenic Disorder | 178,920 |
| S3 | S1 OR S2 | 178,920 |
| S4 | (MH "Positive Psychology") | 189 |
| S5 | Psychology, Positive | 185,401 |
| S6 | S4 OR S5 | 185,401 |
| S7 | (MH "Randomized Controlled Trials") | 174,380 |
| S8 | randomized controlled trial OR randomized OR randomly OR trial | 2,176,702 |
| S9 | S7 OR S8 | 2,176,702 |
| S10 | S3 AND S6 AND S9 | 1,785 |
|  | **Cochrane Library** |  |
| #1 | (Schizophrenia):ti,ab,kw or (Schizophrenias):ti,ab,kw or (Dementia Praecox):ti,ab,kw or (Schizophrenic Disorders):ti,ab,kw or (Disorder, Schizophrenic):ti,ab,kw or (Disorders, Schizophrenic):ti,ab,kw or (Schizophrenic Disorder):ti,ab,kw or (schizophrenic syndrome):ti,ab,kw | 20298 |
| #2 | (Psychology, Positive):ti,ab,kw or (Positive Psychology):ti,ab,kw | 13919 |
| #3 | (randomized controlled trial):ti,ab,kw or (randomized):ti,ab,kw or (randomly):ti,ab,kw or (trial):ti,ab,kw | 1486635 |
| #4 | #1 and #2 and #3 | 1009 |
