# Supplementary material for: The effects of positive psychology theory in the rehabilitation nursing of Chinese patients with schizophrenia: a systematic review and meta-analysis of randomized controlled trials
Source: Front Psychiatry. 2025 Feb 19;16:1515028. doi: 10.3389/fpsyt.2025.1515028 (PMC11880031; doi:10.3389/fpsyt.2025.1515028)
Supplement: Supplementary file 1 [file DataSheet1.zip › Data sheet 1/S2 GRADE Assessment.docx.docx]

**Table GRADE Assessment**

| Outcomes | Study  Design | Risk of  Bias | Inconsistency | Indirectness | Imprecision | Other  Considerations | No. of Participants | | Effect(95%CI) | Quality |
| --- | --- | --- | --- | --- | --- | --- | --- | --- | --- | --- |
|  |  |  |  |  |  |  | positive psychology | Control |  |  |
| Mental health | RCTs | Serious | Serious | No | No | No | 1047 | 1043 | MD=43.50(40.11 to 46.89) | Low |
| Well-being | RCTs | Serious | No | No | No | No | 827 | 823 | MD=0.61(0.56 to 0.66) | Moderate |
| Social function | RCTs | Serious | Serious | No | No | Serious | 688 | 684 | SMD=-2.68(-3.26 to -2.10) | Very low |
| Social adaptability | RCTs | Serious | No | No | Serious | No | 204 | 204 | MD=-8.72(-9.16 to -8.27) | Low |
| Positive Symptom | RCTs | Serious | No | No | Serious | No | 431 | 431 | SMD=-2.68(-3.53 to -1.84) | Low |
| Negative Symptom | RCTs | Serious | No | No | Serious | No | 391 | 391 | SMD=-2.63(-3.40 to -1.87) | Low |
| PANSS-Total | RCTs | Serious | No | No | Serious | No | 273 | 273 | SMD=-1.99(-2.20 to -1.78) | Low |
| Cognitive function | RCTs | Serious | No | No | Serious | No | 182 | 182 | MD=2.38(1.97 to 2.78) | Low |
| Self-esteem | RCTs | Serious | No | No | Serious | No | 254 | 254 | MD=7.98(7.53 to 8.42) | Low |

**Positive psychology theory versus TAU**
