# Supplementary figures and images for: The effects of positive psychology theory in the rehabilitation nursing of Chinese patients with schizophrenia: a systematic review and meta-analysis of randomized controlled trials
Source: Front Psychiatry. 2025 Feb 19;16:1515028. doi: 10.3389/fpsyt.2025.1515028 (PMC11880031; doi:10.3389/fpsyt.2025.1515028)

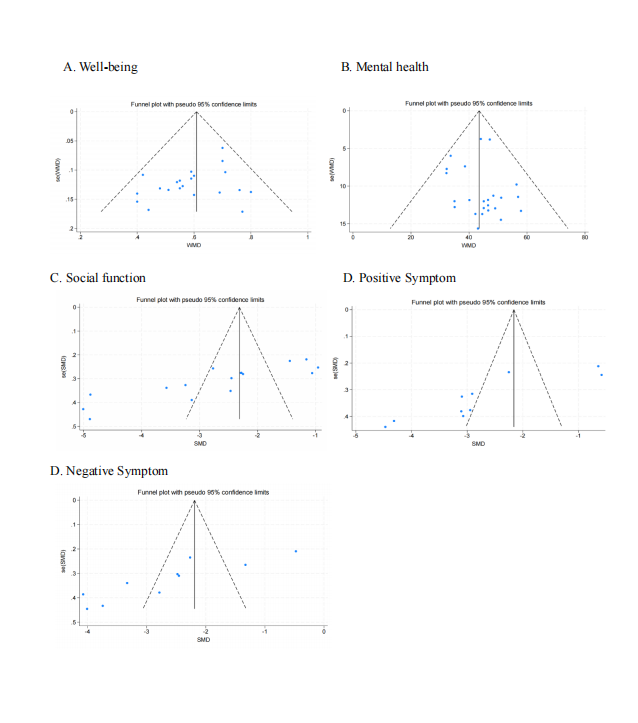

Supplement: Supplementary file 1 [file DataSheet1.zip › Data sheet 1/S10 Publication bias assessment funnel plot.png]

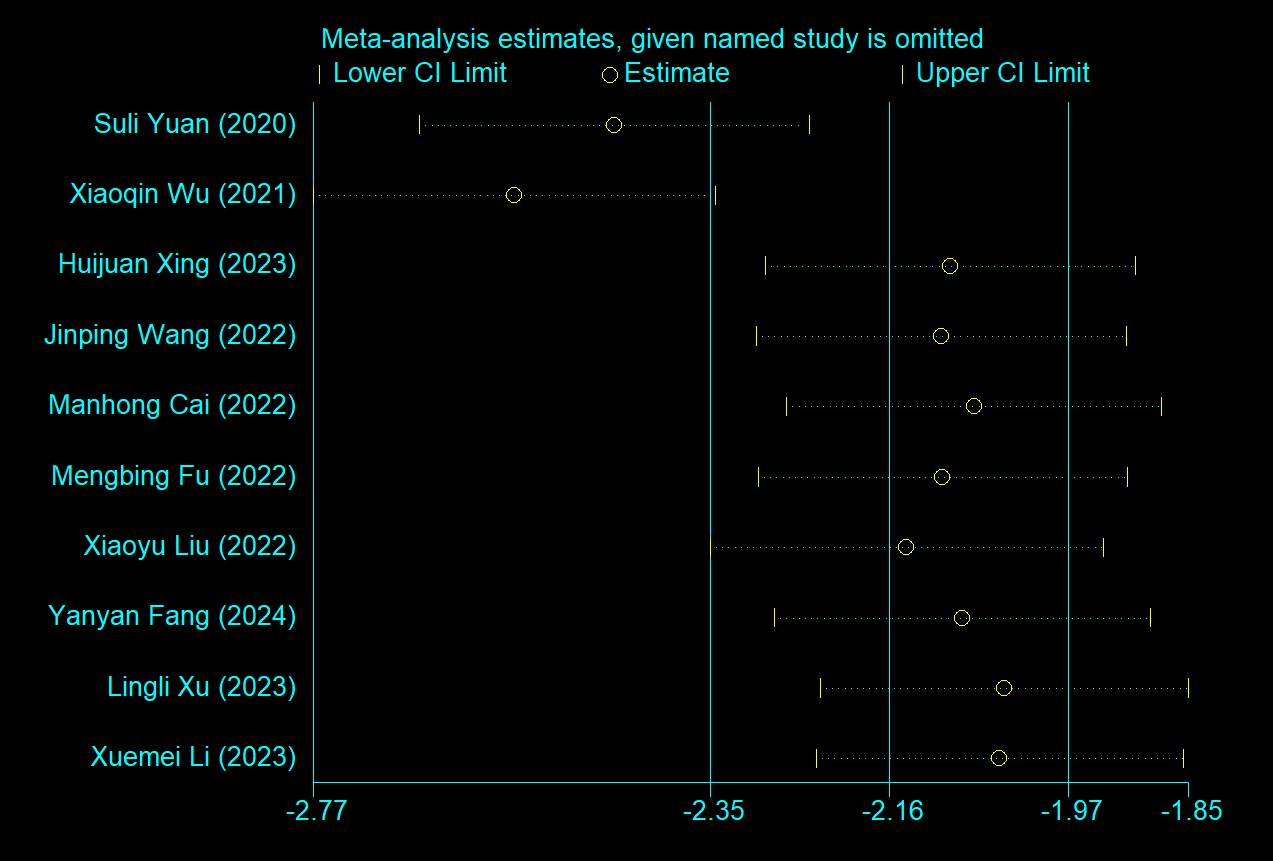

Supplement: Supplementary file 1 [file DataSheet1.zip › Data sheet 1/S4 Positive Symptom Sensitivity Analysis Chart.png]

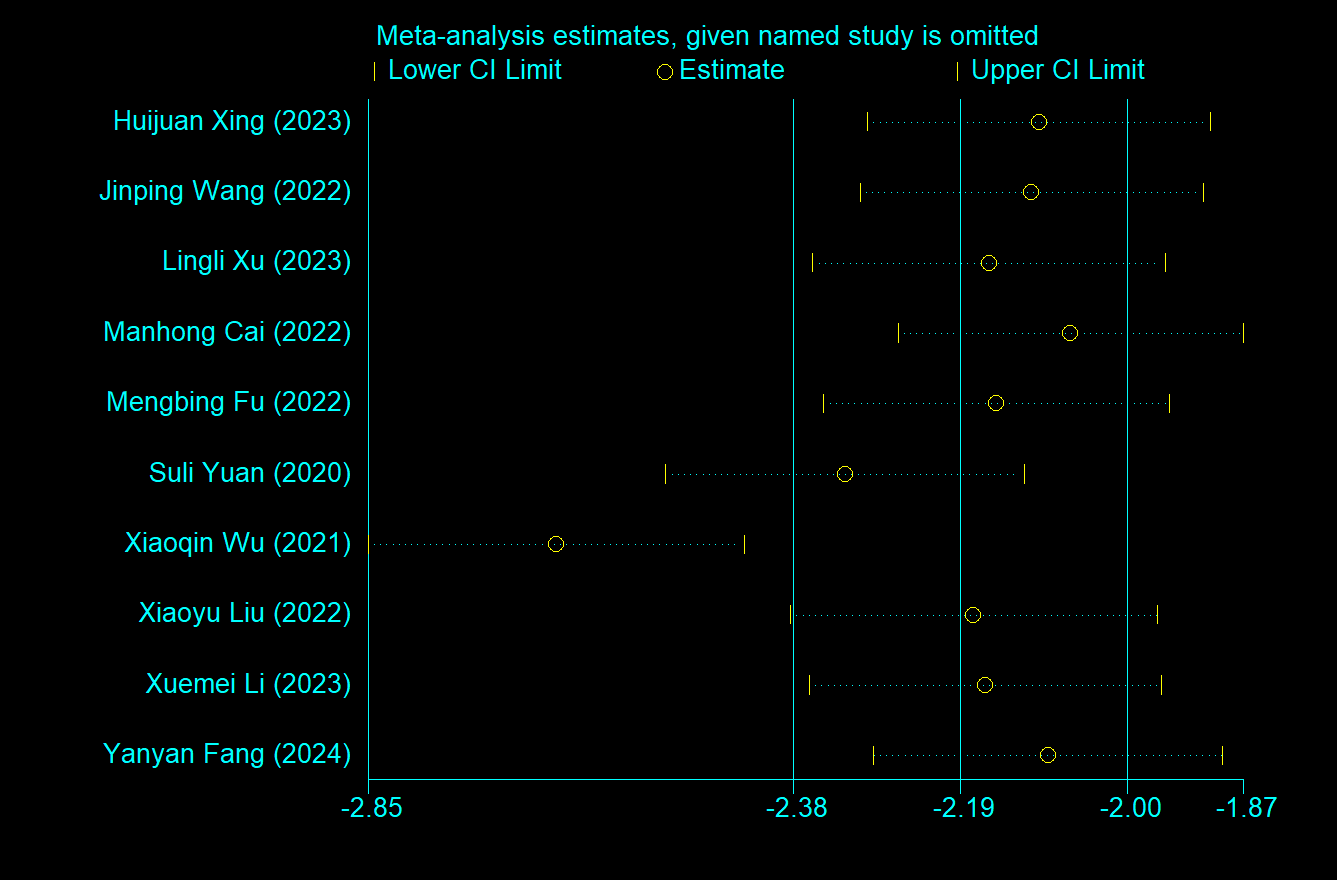

Supplement: Supplementary file 1 [file DataSheet1.zip › Data sheet 1/S5 Negative Symptom Sensitivity Analysis Chart.png]

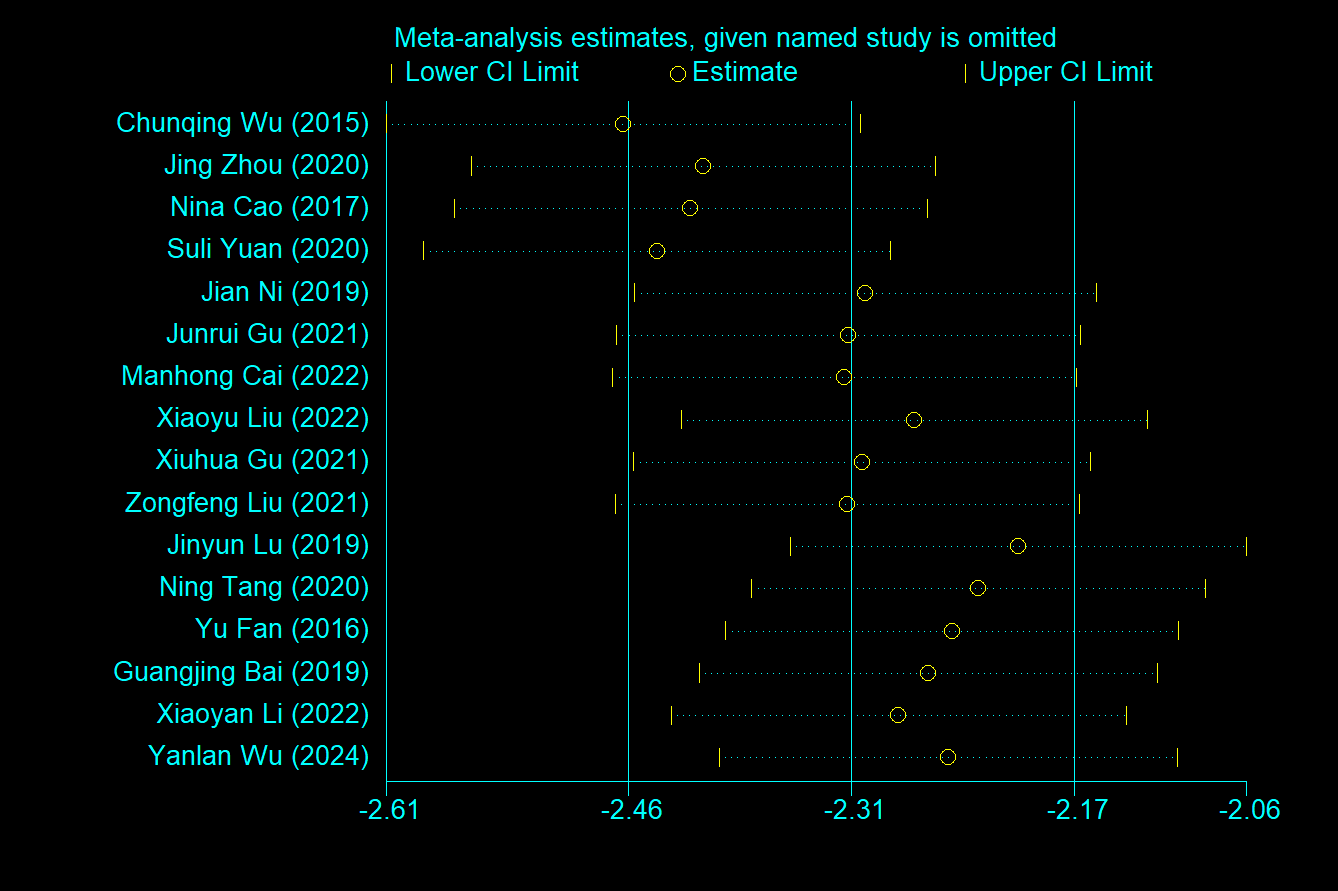

Supplement: Supplementary file 1 [file DataSheet1.zip › Data sheet 1/S6 Social Functioning Sensitivity Analysis Chart.png]

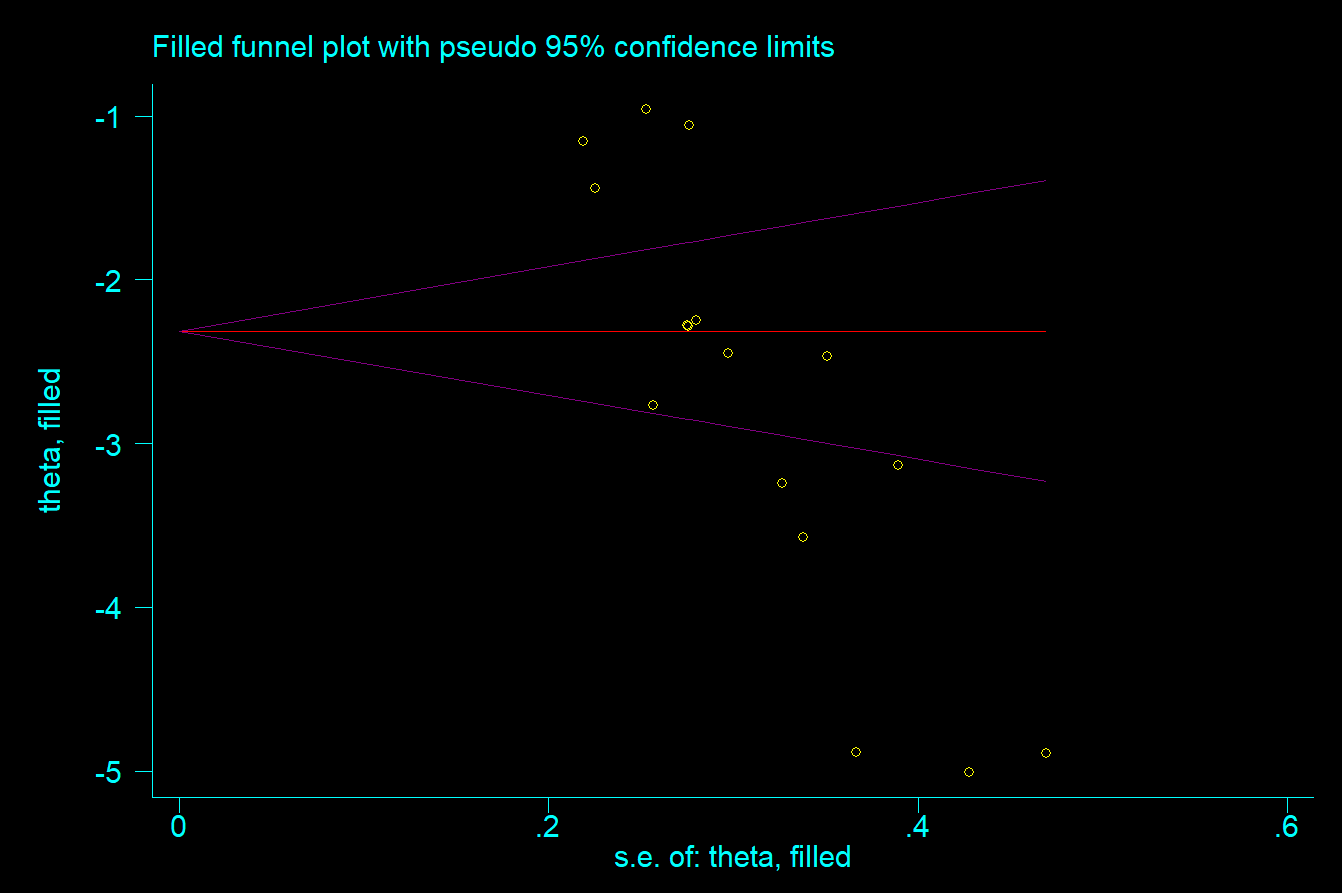

Supplement: Supplementary file 1 [file DataSheet1.zip › Data sheet 1/S7 Trim and Fill Plot of Social Function.png]

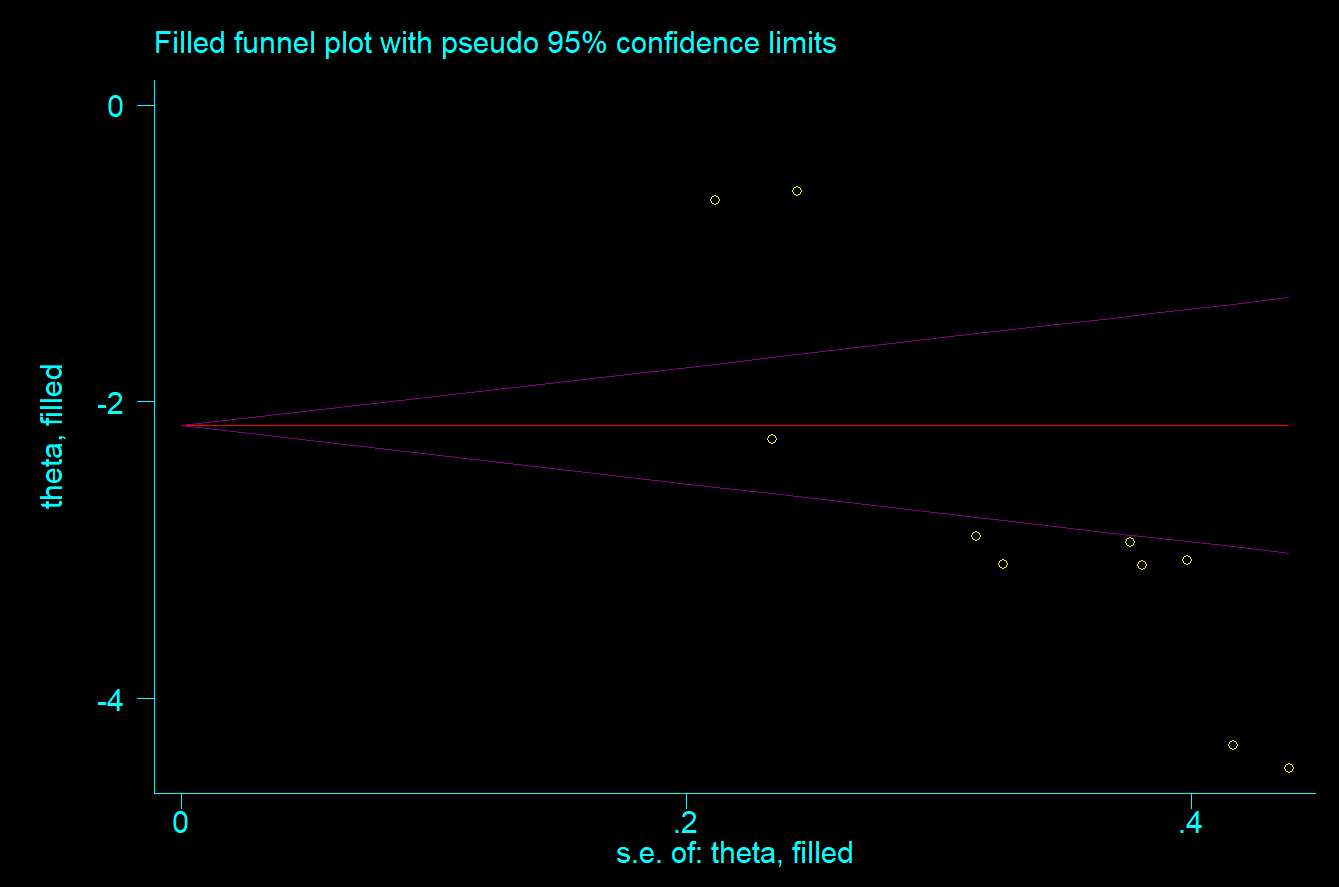

Supplement: Supplementary file 1 [file DataSheet1.zip › Data sheet 1/S8 Trim and Fill Plot of Positive Symptom.png]

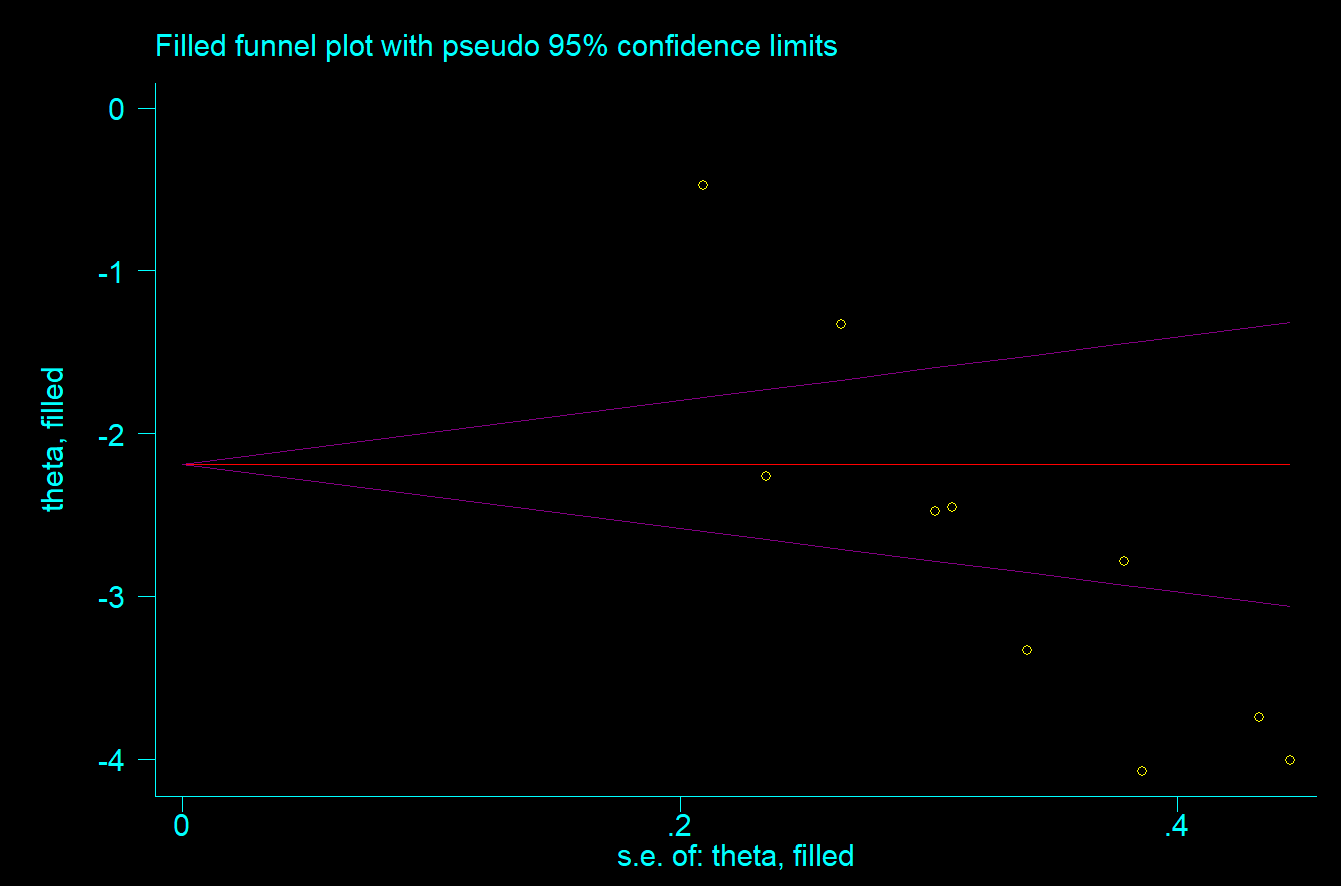

Supplement: Supplementary file 1 [file DataSheet1.zip › Data sheet 1/S9 Trim and Fill Plot of Negative Symptom.png]
